# Identification and characterization of the BRI2 interactome in the brain

**DOI:** 10.1038/s41598-018-21453-3

**Published:** 2018-02-23

**Authors:** Filipa Martins, Ana M. Marafona, Cátia D. Pereira, Thorsten Müller, Christina Loosse, Katharina Kolbe, Odete A. B. da Cruz e Silva, Sandra Rebelo

**Affiliations:** 1 Neuroscience and Signalling Laboratory, Department of Medical Sciences, Institute of Biomedicine-iBiMED, University of Aveiro, Aveiro, Portugal; 2 Leibniz-Institut für Analytische Wissenschaften -ISAS- e. V., Dortmund, Germany; 3 Cell Signaling, Department of Molecular Biochemistry, Faculty of Chemistry and Biochemistry, Ruhr University Bochum, Universitätsstr. 150, 44780 Bochum, Germany; 4 Institute of Psychiatric Phenomics and Genomics, Clinical Center of the University of Munich, Nussbaumstr. 7, 80336 Munich, Germany

## Abstract

BRI family proteins are ubiquitous type II transmembrane proteins but BRI2 is highly expressed in some neuronal tissues. Possible BRI2 functions include neuronal maturation and differentiation. Protein complexes appear to be important in mediating its functions. Previously described BRI2 interactors include the Alzheimer’s amyloid precursor protein and protein phosphatase 1, but clearly the identification of novel interactors provides an important tool to understand the role and function of BRI2. To this end three rat brain regions (cerebellum, hippocampus, and cerebral cortex) were processed by BRI2 immunoprecipitation; co-precipitating proteins were identified by Nano-HPLC-MS/MS. The pool of the brain regions resulted in 511 BRI2 interacting proteins (BRI2 brain interactome) of which 120 were brain specific and 49 involved in neuronal differentiation. Brain region-specific analyses were also carried out for cerebellum, hippocampus, and cerebral cortex. Several novel BRI2 interactors were identified among them DLG4/PSD-95, which is singularly important as it places BRI2 in the postsynaptic compartment. This interaction was validated as well as the interaction with GAP-43 and synaptophysin. In essence, the resulting BRI2 brain interactome, associates this protein with neurite outgrowth and neuronal differentiation, as well as synaptic signalling and plasticity. It follows that further studies should address BRI2 particularly given its relevance to neuropathological conditions.

## Introduction

BRI2 (also known as ITM2B, integral membrane protein 2B) is a ubiquitously expressed type II transmembrane protein that belongs to a family comprising two additional members, BRI1 and BRI3. BRI2 mRNA was found highly expressed in the brain, placenta, pancreas, and kidney, whereas lower expression levels were observed in heart, lung, liver and skeletal muscle. Remarkably, in the brain, BRI2 is predominantly detected in the cerebellum, spinal cord, subthalamic nucleus, substantia nigra, and hippocampus. However, lower expression levels were detected in the cerebral cortex, amygdala and thalamus^1^. Moreover, using *in situ* hybridization it was demonstrated that within the brain, BRI2 mRNA is distributed in different cellular populations, namely in neurons, astrocytes, and microglial cells as well as in smooth muscle and cerebral endothelial cells^2^. Subcellularly, BRI2 is mainly localized in the endoplasmic reticulum (ER), Golgi apparatus, plasma membrane and cytoplasmic vesicles^3^. BRI2 undergoes regulated intramembrane proteolysis (RIP) in the cis- or medial-Golgi resulting in the formation of several secreted peptides, including the C-terminal 23-residue peptide, the BRICHOS domain, the NTF and BRI2 C-terminal domain, and an intracellular domain (BRI2ICD), as consequence of the activity of several proteases^3^.

The distribution of BRI2 within proximal dendrites and axons, as well as cell bodies, and its presence in some neuropathological structures like dystrophic neurites, suggests that BRI2 could be anterogradely transported to the nerve terminals, where it may have a role^4^. In fact, BRI2 overexpression in human neuronal cells induces neurites’ elongation, indicating that BRI2 could be involved in neurite outgrowth^3,5^. Moreover, BRI2 expression increases during neuronal maturation and differentiation suggesting an important role for BRI2 both during neuronal development but also in the adult brain^6^. In addition, some BRI2 binding proteins have been identified; as is the case with the Alzheimer’s amyloid precursor protein (APP). BRI2:APP interaction occurs at the cell surface and in endocytic compartments, and this complex could potentially regulate APP processing and inhibit Abeta production^7,8^. Interestingly, BRI2 accumulates in the hippocampus in the early stages of Alzheimer’s disease (AD) preventing the BRI2:APP complex formation and therefore resulting in increased APP processing and consequently more Abeta_1-40_ and Abeta_1-42_ is produced^9^. Additionally, we recently reported that BRI2 interacts with protein phosphatase 1 (PP1) via a well conserved RVxF motif (^3^KVTF^6^) located at the BRI2 N-terminal domain. Further, we established that BRI2 is dephosphorylated by this protein phosphatase; given that when the BRI2:PP1 interaction is abolished, BRI2 phosphorylation levels increase dramatically. The morphological consequence of inhibiting PP1 binding was a phenotype consistent with neurite outgrowth and neuronal differentiation^5,6^.

In addition to AD, the clinical relevance of this protein lies in two different autosomal dominant mutations in the *ITM2B* gene, which are associated with two rare early-onset forms of dementia, the Familial British and Danish dementias (FBD and FDD, respectively). These diseases share several clinical symptoms, such as progressive cognitive impairment cerebellar ataxia and spasticity^10^. Neuropathological hallmarks of FBD consist in the presence of pre-amyloid and amyloid parenchymal lesions in the brain (primarily localized to the hippocampus and cerebellum), extensive cerebral amyloid angiopathy (CAA) and intraneuronal formation of neurofibrillary tangles (NFT) within the limbic regions^11,12^. Neuropathological lesions in FDD patients are closely similar to those found in FBD, however in FDD co-deposition of Abeta and Danish amyloid is observed, but parenchymal compact plaques are absent^13,14^.

To date, the precise physiological BRI2 function is not fully elucidated. Even so several lines of evidence strengthen the association of BRI2 with neuronal functions, in particular, the identification and functional characterization of BRI2 interactors, namely APP and PP1. Therefore, the identification of novel BRI2 interactors that may play important roles in regulating its trafficking, processing and signalling effect is an essential point. Additionally, specific cellular signalling pathways and processes which are crucial to the understanding of the physiological and pathological role of BRI2, in particular in the brain, may be forthcoming. The work here described proposes novel BRI2 interactors in rat brain, which represent a valuable tool to study the brain function in mammals. BRI2 interactors in three different brain regions were investigated; the cerebellum, hippocampus, and cerebral cortex, followed by *in silico* analysis, such as Gene Ontology (GO), biological pathways, and protein-protein interaction (PPI) network analysis. This permitted identifying potentially novel BRI2 functional relationships via analysing its interacting proteins in the brain.

## Results

### Identification of BRI2 brain interactome

**Figure 1:**
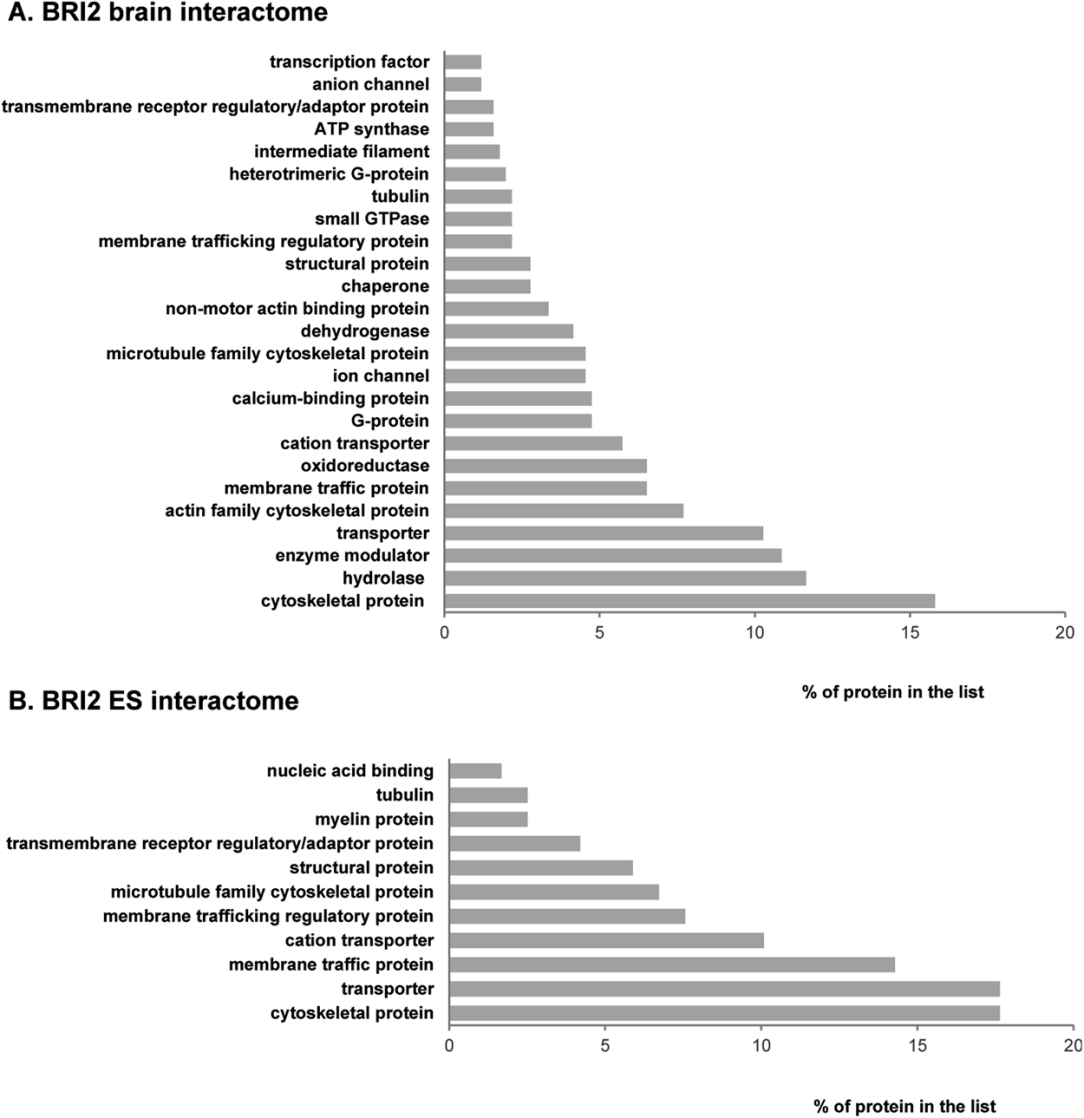
Panther Protein class of the novel BRI2 interactome using the PANTHER online resource. The percentage of the BRI2 interacting proteins in each category was calculated and plotted. (**A**) Panther Protein class of all the identified BRI2 interacting proteins (BRI2 brain interactome). (**B**) Panther Protein class of the identified brain-enriched or specific BRI2 interacting proteins (BRI2 brain ES interactome).

Given that BRI2 has been recently associated with important neuronal functions, the identification of BRI2 brain interactors is of paramount importance. Therefore, our main goal was the identification of novel putative BRI2 interacting proteins in rat brain. Given the expression pattern of BRI2 in the brain, three different regions were chosen, namely cerebral cortex, hippocampus and cerebellum, and the workflow carried out is summarized and presented in Supplementary Figure S1. Briefly, the above-mentioned brain regions were dissected out and subjected to co-immunoprecipitation (co-IP) using a BRI2 specific antibody against the N-terminal region of the protein. For the co-IP experiments, two negative controls were used for each specific brain region. For one of the control Dynabeads Protein G in the absence of the BRI2 antibody was added to the tissue lysates, while in the second control Dynabeads Protein G was conjugated with anti-mouse IgGs. The co-IPed proteins were analyzed by HPLC-MS/MS. The identified proteins were subjected to data processing and filtering; some proteins were only identified in the BRI2 co-IP samples and never in the controls, while others appeared both in the controls IPs and BRI2 co-IPs. These latter proteins were considered as BRI2 putative interactors if the peptide spectrum matches (PSM) ratio between co-IP samples and controls ≥ 2.0. Overall, upon data processing 511 putative BRI2 interacting proteins were identified in the rat brain (Supplementary Table S1). Of note, 342 proteins were identified in the cerebral cortex, 257 in the hippocampus and 262 in the cerebellum (Supplementary Table S1). In Supplementary Table S1, the Uniprot accession numbers are listed as well as the gene and protein name, and the rat brain region in which the peptides were detected. The first analysis of the BRI2 brain interactome (pool of the three brain regions studied) using bioinformatic approaches indicates that the majority of proteins identified in this study belong to the protein classes of cytoskeletal proteins (16%; half of which belong to the actin family), hydrolases (12%), enzyme modulators (11%; mainly G-proteins) and transporters (11%; half of which belong to cation transporters family). Other represented protein classes included membrane traffic proteins (7%), oxidoreductases (7%), calcium-binding proteins (5%), ion channels (5%; being 2% anion channels), dehydrogenases (5%), chaperones (3%), structural proteins (3%), ATP synthases (2%), transmembrane receptor regulatory/adaptor proteins (2%) and transcription factors (1%) (Fig. 1A and Supplementary Table S2).

In parallel, a curated list of 45 proteins that were previously identified as putative BRI2 interacting proteins was retrieved, as described in methods section (Supplementary Table S3). Overall, most of these proteins were identified by yeast-two hybrid and high-throughput screens and were not further validated by additional assays. Of note, in the study here described, 2 of the 45 previously reported BRI2 interacting proteins were also identified: the dihydropyridine-sensitive L-type, calcium channel alpha-2/delta subunit (Cacna2d1) and the serine/threonine-protein phosphatase PP1-alpha catalytic subunit (Ppp1ca).

### Highly enriched and specific brain BRI2 interacting proteins

Given that the main goal of this work is to unravel putative associations of BRI2 to specific cellular functions as well as pathways where BRI2 participates in the brain related processes, a tissue-specific/enriched network approach was employed. For this purpose, the enriched and specific brain proteins from the candidate BRI2 pooled interactome (cerebral cortex + hippocampus + cerebellum) using three different databases were retrieved, as explained in the materials and methods section. From the 511 putative BRI2 interacting proteins identified by Nano-HPLC-MS/MS, a total of 120 proteins are highly enriched/specifically (ES) expressed in the brain (BRI2 ES interactome) (Table 1).

**Table 1:** Novel candidate BRI2 interacting proteins highly enriched or specific for the brain tissue. Uniprot accession numbers, gene and protein names, are listed, as well as the rat brain tissues where the proteins were found. CC, cerebral cortex; HP, hippocampus; CB, cerebellum.

Uniprot accession	Gene name	Protein name	Brain regions
P13233	*Cnp*	2′,3′-cyclic-nucleotide 3′-phosphodiesterase	CC, HP, CB
P23565	*Ina*	Alpha-internexin	CC, HP, CB
O08838	*Amph*	Amphiphysin	CC, HP, CB
P0C6S7	*Anks1b*	Ankyrin repeat and sterile alpha motif domain-containing protein 1B	CC
Q8CGU4	*Agap2*	Arf-GAP with GTPase, ANK repeat and PH domain-containing protein 2	CC
Q6PST4	*Atl1*	Atlastin-1	CC
Q05764	*Add2*	Beta-adducin	HP
P85969	*Napb*	Beta-soluble NSF attachment protein	HP
Q05175	*Basp1*	Brain acid soluble protein 1	HP
P55068	*Bcan*	Brevican core protein	HP, CB
P11275	*Camk2a*	Calcium/calmodulin-dependent protein kinase type II subunit alpha	CC, HP
P08413	*Camk2b*	Calcium/calmodulin-dependent protein kinase type II subunit beta	CC, HP, CB
Q62717	*Cadps*	Calcium-dependent secretion activator 1	HP
Q63092	*Camkv*	CaM kinase-like vesicle-associated protein	HP
Q1WIM1	*Cadm4*	Cell adhesion molecule 4	CB
Q5FVI4	*Cend1*	Cell cycle exit and neuronal differentiation protein 1	CC, HP
Q05140	*Snap91*	Clathrin coat assembly protein AP180	CC, HP
P63041	*Cplx1*	Complexin-1	HP
Q9Z1T4	*Cnksr2*	Connector enhancer of kinase suppressor of ras 2	CC
Q63198	*Cntn1*	Contactin-1	CC, HP
P97846	*Cntnap1*	Contactin-associated protein 1	CC, CB
Q5BJS7	*Cpne9*	Copine-9	HP
Q62950	*Crmp1*	Dihydropyrimidinase-related protein 1	HP
Q62951	*Dpysl4*	Dihydropyrimidinase-related protein 4	CB
Q63622	*Dlg2*	Disks large homolog 2	CC
P31016	*Dlg4*	Disks large homolog 4	CC, HP, CB
P97836	*Dlgap1*	Disks large-associated protein 1	CC
P97837	*Dlgap2*	Disks large-associated protein 2	CC
P97838	*Dlgap3*	Disks large-associated protein 3	CC
P21575	*Dnm1*	Dynamin-1	CC, HP
Q8R491	*Ehd3*	EH domain-containing protein 3	HP
Q8CH84	*Elavl2*	ELAV-like protein 2	CC
O35179	*Sh3gl2*	Endophilin-A1	CC, CB
P24942	*Slc1a3*	Excitatory amino acid transporter 1	CC, HP, CB
P31596	*Slc1a2*	Excitatory amino acid transporter 2	CC, HP, CB
O35921	*Slc1a6*	Excitatory amino acid transporter 4	CB
O88871	*Gabbr2*	Gamma-aminobutyric acid type B receptor subunit 2	CC
P47819	*Gfap*	Glial fibrillary acidic protein	CC, HP, CB
P19490	*Gria1*	Glutamate receptor 1	CC
P19491	*Gria2*	Glutamate receptor 2	CC, HP, CB
P35439	*Grin1*	Glutamate receptor ionotropic, NMDA 1	CC
Q00960	*Grin2b*	Glutamate receptor ionotropic, NMDA 2B	CC
Q9WTT6	*Gda*	Guanine deaminase	CC
P19627	*Gnaz*	Guanine nucleotide-binding protein G	CC, HP
P59215	*Gnao1*	Guanine nucleotide-binding protein G	CC, HP, CB
Q9Z214	*Homer1*	Homer protein homolog 1	CC
Q9ESM2	*Hapln2*	Hyaluronan and proteoglycan link protein 2	CC, CB
Q9QYU4	*Crym*	Ketimine reductase mu-crystallin	HP
Q6QLM7	*Kif5a*	Kinesin heavy chain isoform 5 A	CC, HP, CB
P56536	*Kif5c*	Kinesin heavy chain isoform 5 C	CC, CB
P37285	*Klc1*	Kinesin light chain 1	CC, HP, CB
Q62813	*Lsamp*	Limbic system-associated membrane protein	CC, CB
P34926	*Map1a*	Microtubule-associated protein 1 A	CC, HP
P15205	*Map1b*	Microtubule-associated protein 1B	CC, HP, CB
P15146	*Map2*	Microtubule-associated protein 2	CC
Q63560	*Map6*	Microtubule-associated protein 6	CC, HP, CB
Q505J6	*Slc25a18*	Mitochondrial glutamate carrier 2	CC
P02688	*Mbp*	Myelin basic protein	CC
P60203	*Plp1*	Myelin proteolipid protein	CC, CB
P07722	*Mag*	Myelin-associated glycoprotein	CC, CB
Q63345	*Mog*	Myelin-oligodendrocyte glycoprotein	CC, CB
P13596	*Ncam1*	Neural cell adhesion molecule 1	CB
P55067	*Ncan*	Neurocan core protein	CC, HP, CB
O35095	*Ncdn*	Neurochondrin	CC
P19527	*Nefl*	Neurofilament light polypeptide	CC, HP, CB
P12839	*Nefm*	Neurofilament medium polypeptide	CC, CB
P07936	*Gap43*	Neuromodulin	CC
Q9ESI7	*Dcx*	Neuronal migration protein doublecortin	CC
Q9WU34	*Sept3*	Neuronal-specific septin-3	HP
Q62718	*Ntm*	Neurotrimin	CC, CB
P11506	*Atp2b2*	Plasma membrane calcium-transporting ATPase 2	CC
Q64568	*Atp2b3*	Plasma membrane calcium-transporting ATPase 3	HP
P10499	*Kcna1*	Potassium voltage-gated channel subfamily A member 1	CC
Q6MG82	*Prrt1*	Proline-rich transmembrane protein 1	HP
O88778	*Bsn*	Protein bassoon	CC, CB
P63319	*Prkcg*	Protein kinase C gamma type	CC, HP, CB
Q9JKS6	*Pclo*	Protein piccolo	CC
P47709	*Rph3a*	Rabphilin-3A	CC, HP
P63012	*Rab3a*	Ras-related protein Rab-3A	CC, HP, CB
P62824	*Rab3c*	Ras-related protein Rab-3C	CC, HP
Q9JIR4	*Rims1*	Regulating synaptic membrane exocytosis protein 1	CC
Q64548	*Rtn1*	Reticulon-1	CC, HP, CB
Q9JKE3	*Scamp5*	Secretory carrier-associated membrane protein 5	CC, HP
Q9JJM9	*Sept5*	Septin-5	CC, HP
O08875	*Dclk1*	Serine/threonine-protein kinase DCLK1	CC, HP
Q9WV48	*Shank1*	SH3 and multiple ankyrin repeat domains protein 1	CC, CB
P0DJJ3	*Sgip1*	SH3-containing GRB2-like protein 3-interacting protein 1	CC, HP
P31647	*Slc6a11*	Sodium- and chloride-dependent GABA transporter 3	CC, CB
P48768	*Slc8a2*	Sodium/calcium exchanger 2	CC
P06686	*Atp1a2*	Sodium/potassium-transporting ATPase subunit alpha-2	CC
P06687	*Atp1a3*	Sodium/potassium-transporting ATPase subunit alpha-3	CC, HP
P13638	*Atp1b2*	Sodium/potassium-transporting ATPase subunit beta-2	CC, HP, CB
Q63633	*Slc12a5*	Solute carrier family 12 member 5	HP
Q9QWN8	*Sptbn2*	Spectrin beta chain, non-erythrocytic 2	CC, CB
Q9QXY2	*Srcin1*	SRC kinase signaling inhibitor 1	CC, CB
P09951	*Syn1*	Synapsin-1	CC, HP
Q63537	*Syn2*	Synapsin-2	CC
Q02563	*Sv2a*	Synaptic vesicle glycoprotein 2 A	CC, HP, CB
Q63564	*Sv2b*	Synaptic vesicle glycoprotein 2B	CC, HP
P07825	*Syp*	Synaptophysin	CC, HP
P60881	*Snap25*	Synaptosomal-associated protein 25	HP, CB
P21707	*Syt1*	Synaptotagmin-1	CC, HP, CB
P97610	*Syt12*	Synaptotagmin-12	CC, CB
P29101	*Syt2*	Synaptotagmin-2	CB
Q62747	*Syt7*	Synaptotagmin-7	CB
P32851	*Stx1a*	Syntaxin-1A	CC
P61265	*Stx1b*	Syntaxin-1B	CC, HP, CB
P61765	*Stxbp1*	Syntaxin-binding protein 1	CC, HP
Q05546	*Tnr*	Tenascin-R	CC, HP, CB
P70566	*Tmod2*	Tropomodulin-2	CC, CB
P85108	*Tubb2a*	Tubulin beta-2A chain	CC, HP, CB
Q3KRE8	*Tubb2b*	Tubulin beta-2B chain	CC, HP
Q4QRB4	*Tubb3*	Tubulin beta-3 chain	CC, HP, CB
Q9QUL6	*Nsf*	Vesicle-fusing ATPase	CC, HP, CB
Q62634	*Slc17a7*	Vesicular glutamate transporter 1	CC, HP
Q9JI12	*Slc17a6*	Vesicular glutamate transporter 2	CC
O35458	*Slc32a1*	Vesicular inhibitory amino acid transporter	CC, HP
P62762	*Vsnl1*	Visinin-like protein 1	CB
Q71RJ2	*Cacng2*	Voltage-dependent calcium channel gamma-2 subunit	CB
Q6QIX3	*Slc30a3*	Zinc transporter 3	CC

Almost half of these interactors are cytoskeletal proteins (around 18%; mainly actin and microtubules) or transporters (around 18%; half of which belong to cation transporters family), followed by membrane traffic proteins (14%; half of which are regulatory), structural proteins (6%: half of which are myelin proteins), transmembrane receptor regulatory/adaptor proteins (3%) and nucleic acid binding proteins (2%) (Fig. 1B and Supplementary Table S4).

**Figure 2:**
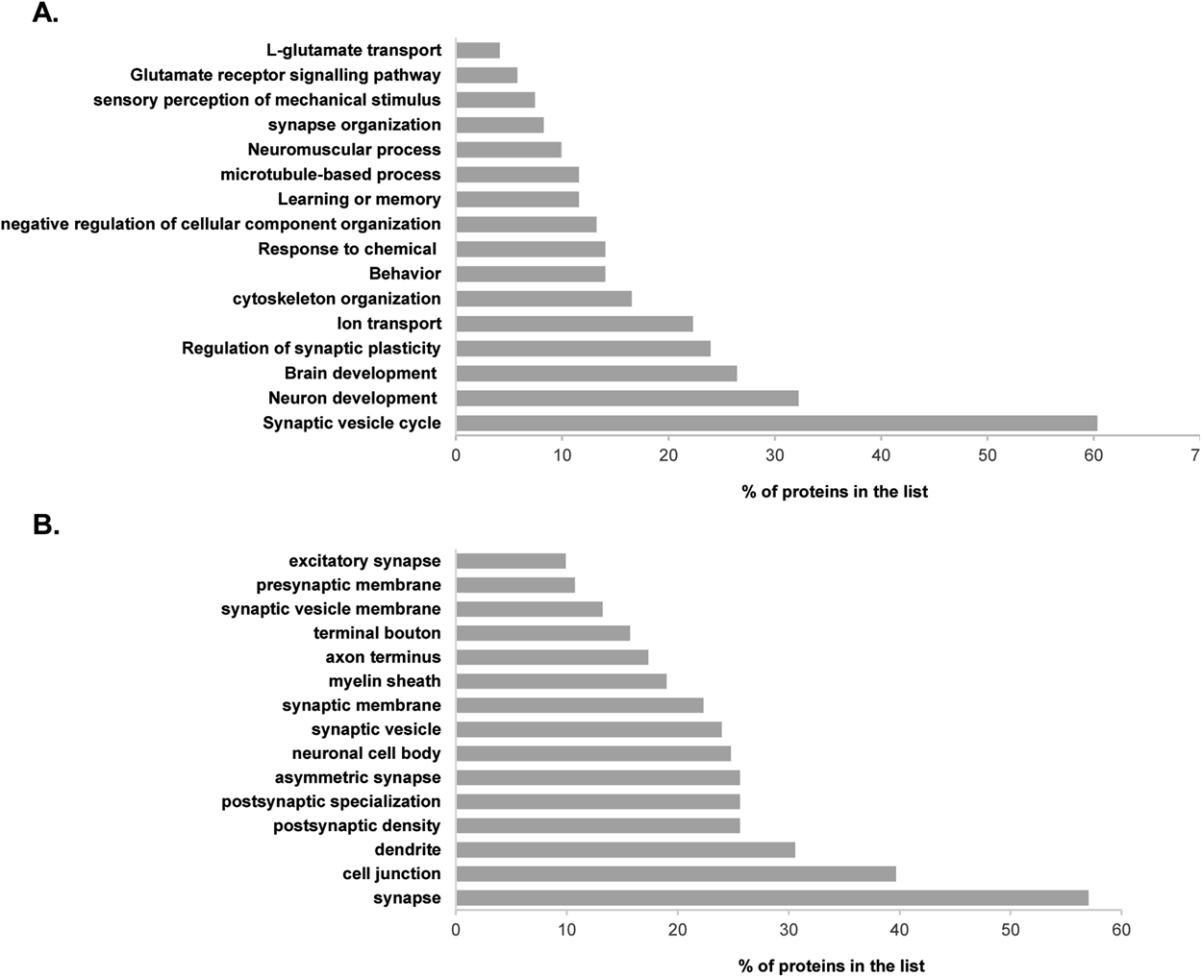
Functional enrichment analysis of the BRI2 brain ES interactome using the PANTHER online resource. The percentage of the BRI2 interacting proteins in each category was calculated and plotted. (**A**) Biological process analysis of the identified BRI2 interacting proteins particularly brain-enriched or specific. (**B**) Cellular component analysis of the identified BRI2 interacting proteins particularly brain-enriched or specific.

In order to elucidate if the proteins identified in BRI2 ES interactome could be assigned to specific functions, and ultimately to provide more information regarding the physiological role of BRI2, particularly in the brain, a further classification was performed into functional categories according to Gene Ontology (GO) annotation using the PANTHER online resource. Regarding the analysis of the over-represented biological process GO terms (Fig. 2A and Supplementary Table S5), the results revealed that the BRI2 ES interactome included proteins involved in several functional roles, from which the synaptic vesicle cycle (60.3%), neuron development (32.2%), brain development (26.4%), regulation of synaptic plasticity (24.0%), ion transport (22.3%), and cytoskeleton organization (16.5%) stand out (Fig. 2A). Cellular component annotation analysis revealed several statistically enriched categories (Supplementary Table S6). Of note, a high percentage of proteins in the BRI2 ES interactome that localized to the synapse (57.0%), dendrite (30.6%), postsynaptic density (25.6%), synaptic vesicle (24.0%), axon terminus (17.4%) and presynaptic membrane (10.7%) were found (Fig. 2B).

### BRI2 role in neuronal differentiation

Previous results strongly suggested a role for BRI2 in neuronal differentiation^4–6^, however to date the exact mechanisms or pathways involving BRI2 have not been revealed. Hence from the BRI2 brain ES interactors, the proteins annotated with GO terms related to neuronal differentiation were retrieved. This resulted in a list of 49 proteins corresponding to the BRI2 ES neuronal differentiation interactome (Supplementary Table S7). To evaluate the interconnection of these BRI2 candidate interactors, a PPI network augmentation was performed, using the Cytoscape software^15^, which enhanced the experimental data with additional known and experimentally validated PPI from public databases (see materials and methods section). In addition, the proteins annotated with GO terms related to neuronal differentiation from the previously identified list of BRI2 interactors (Supplementary Table S3), were manually added to this PPI network. The PPI network augmentation provides interesting and valuable information, permitting a systems approach to analyze the complexes formed from the co-immunoprecipitating proteins rather than a single interaction.

**Figure 3:**
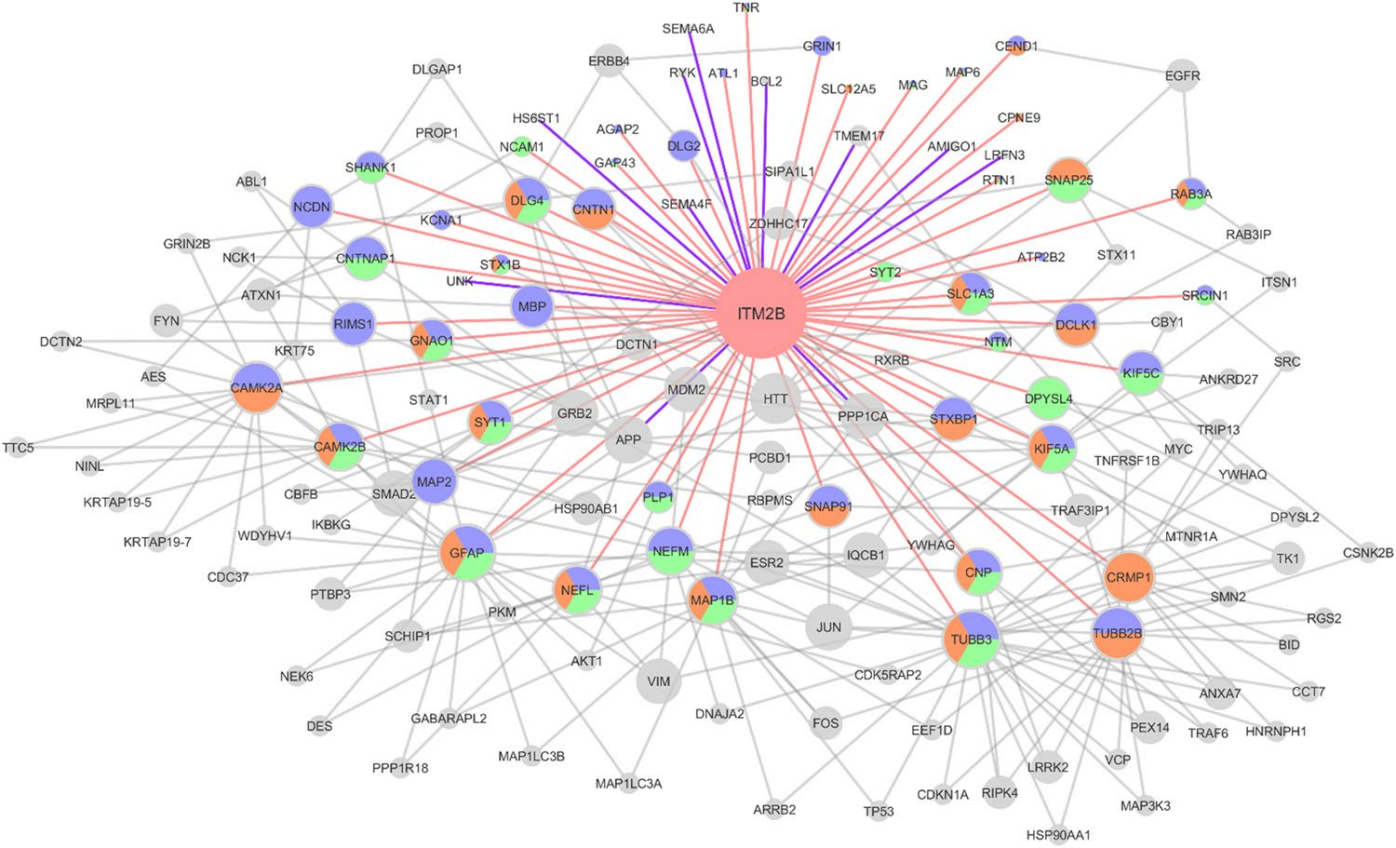
BRI2 brain ES sub-network for neuronal differentiation annotated proteins. Node colors represent the source of the protein: blue nodes correspond to proteins identified in this study in the cerebral cortex, orange nodes correspond to proteins identified in this study in the hippocampus, green nodes correspond to proteins identified in this study in the cerebellum, and grey nodes are proteins added by network augmentation. Node size according to the degree in the network. Edge color represents the source of interaction: light red edges correspond to the novel BRI2 interactions identified in our study, whereas the grey edges correspond to interactions added by network augmentation.

The network construction and analysis were achieved using the NetworkAnalyzer plugin of the Cytoscape^15^. In the resulting BRI2 (gene *ITM2B*) PPI network (Fig. 3), the grey nodes correspond to PPI added by the Cytoscape analysis, the colored nodes to the proteins identified in this study (blue for proteins identified in cerebral cortex, orange for proteins identified in hippocampus, and green for proteins identified in cerebellum) annotated with GO terms related to neuronal differentiation, and the node size corresponds to its degree (k; the number of edges linked to it). The total number of proteins mapped in the neuronal differentiation-specific PPI network was 144 (nodes), and the total number of connections between them was 286 (edges) (Fig. 3). The average number of neighbors for a node in the network, which indicates the average connectivity of a node in the network, is 3.972. The mean clustering coefficient of the network, which characterizes how nearest neighboring nodes of a node are connected to each other, is 0.09, being 4.5 times larger than the clustering coefficient expected for a sparse random uncorrelated network (0.02)^16,17^.

From all the nodes, the TUBB3 (k = 23) was noteworthy since it presents the highest node degree and in this study, it was identified in the three different brain regions analyzed (Fig. 3). TUBB3 (Tubulin beta-3 chain), is a neuron-specific beta-tubulin which has been suggested to have a critical role in proper axon guidance and maintenance^18^. Additionally, the following proteins were highlighted in the network: the TUBB2B (k = 16), CAMK2A (k = 15), CRMP1 (k = 15), MAP1B (k = 13), CAMK2B (k = 11), NEFL (k = 11), KIF5A (k = 11), NEFM (K = 10) and DLG4 (k = 9) (Fig. 3).

In order to establish the signalling pathways that are most associated with BRI2, an integrated KEGG pathway enrichment analysis of the 144 proteins in BRI2 brain ES neuronal differentiation PPI network was performed using the ClueGo plugin of the Cytoscape software^19^. This analysis placed BRI2 into signalling networks well related to neuronal differentiation, namely ErbB signalling pathway (pValue = 1.812E-08), neurotrophin signalling pathway (pValue = 6.443E-05), estrogen signalling pathway (pValue = 1.163E-05), FoxO signalling pathway (pValue = 5.248E-03), axon guidance (pValue = 1.571E-03), adherens junction (pValue = 2.896E-02) and HIF-1 signalling pathway (pValue = 2.675E-02) (Table 2).

**Table 2:** KEGG category enrichment analysis of the neuronal differentiation annotated network obtained using ClueGo plugin of the Cytoscape software. Enriched categories identified are those with p-value < 0.05.

KEGG Term ID	KEGG Term	Nr. Genes	% Associated Genes	pValue	Associated Genes Found
KEGG:04012	ErbB signaling pathway	12	13.636364	1.81E-08	ABL1, AKT1, CAMK2A, CAMK2B, CDKN1A, EGFR, ERBB4, GRB2, JUN, MYC, NCK1, SRC
KEGG:05215	Prostate cancer	10	11.235955	4.00E-06	AKT1, BCL2, CDKN1A, EGFR, GRB2, HSP90AA1, HSP90AB1, IKBKG, MDM2, TP53
KEGG:05161	Hepatitis B	12	8.219178	6.07E-06	AKT1, BCL2, CDKN1A, FOS, GRB2, IKBKG, JUN, MYC, SRC, STAT1, TP53, YWHAQ
KEGG:05014	Amyotrophic lateral sclerosis (ALS)	8	15.686275	6.07E-06	BCL2, BID, GRIN1, GRIN2B, NEFL, NEFM, TNFRSF1B, TP53
KEGG:04915	Estrogen signaling pathway	10	10.0	1.16E-05	AKT1, EGFR, ESR2, FOS, GNAO1, GRB2, HSP90AA1, HSP90AB1, JUN, SRC
KEGG:05169	Epstein-Barr virus infection	13	6.372549	3.20E-05	AKT1, BCL2, CDKN1A, CSNK2B, IKBKG, JUN, MDM2, MYC, TP53, TRAF6, VIM, YWHAG, YWHAQ
KEGG:05205	Proteoglycans in cancer	13	6.3414636	3.32E-05	AKT1, CAMK2A, CAMK2B, CDKN1A, EGFR, ERBB4, GRB2, MDM2, MYC, PPP1CA, SMAD2, SRC, TP53
KEGG:05214	Glioma	8	12.121212	4.40E-05	AKT1, CAMK2A, CAMK2B, CDKN1A, EGFR, GRB2, MDM2, TP53
KEGG:04722	Neurotrophin signaling pathway	10	8.264462	6.44E-05	ABL1, AKT1, BCL2, CAMK2A, CAMK2B, GRB2, JUN, MAP3K3, TP53, TRAF6
KEGG:05220	Chronic myeloid leukemia	8	10.958904	9.26E-05	ABL1, AKT1, CDKN1A, GRB2, IKBKG, MDM2, MYC, TP53
KEGG:04728	Dopaminergic synapse	10	7.6923075	1.20E-04	AKT1, ARRB2, CAMK2A, CAMK2B, FOS, GNAO1, GRIN2B, KIF5A, KIF5C, PPP1CA
KEGG:05219	Bladder cancer	6	14.634147	3.29E-04	CDKN1A, EGFR, MDM2, MYC, SRC, TP53
KEGG:05210	Colorectal cancer	7	11.290322	3.32E-04	AKT1, BCL2, FOS, JUN, MYC, SMAD2, TP53
KEGG:05031	Amphetamine addiction	7	10.294118	5.92E-04	CAMK2A, CAMK2B, FOS, GRIN1, GRIN2B, JUN, PPP1CA
KEGG:04921	Oxytocin signaling pathway	10	6.289308	6.65E-04	CAMK2A, CAMK2B, CDKN1A, EGFR, FOS, GNAO1, JUN, PPP1CA, RGS2, SRC
KEGG:04360	Axon guidance	10	5.681818	1.57E-03	ABL1, CAMK2A, CAMK2B, DPYSL2, FYN, NCK1, RYK, SEMA4F, SEMA6A, SRC
KEGG:05130	Pathogenic Escherichia coli infection	6	10.909091	1.70E-03	ABL1, FYN, NCK1, TUBB2B, TUBB3, YWHAQ
KEGG:05224	Breast cancer	9	6.1643834	2.00E-03	AKT1, CDKN1A, EGFR, ESR2, FOS, GRB2, JUN, MYC, TP53
KEGG:05222	Small cell lung cancer	7	8.139535	2.46E-03	AKT1, BCL2, IKBKG, MYC, RXRB, TP53, TRAF6
KEGG:04721	Synaptic vesicle cycle	6	9.523809	3.40E-03	RAB3A, RIMS1, SNAP25, STX1B, STXBP1, SYT1
KEGG:04110	Cell cycle	8	6.451613	3.53E-03	ABL1, CDKN1A, MDM2, MYC, SMAD2, TP53, YWHAG, YWHAQ
KEGG:04912	GnRH signaling pathway	7	7.6086955	3.60E-03	CAMK2A, CAMK2B, EGFR, GRB2, JUN, MAP3K3, SRC
KEGG:05212	Pancreatic cancer	6	9.090909	4.12E-03	AKT1, EGFR, IKBKG, SMAD2, STAT1, TP53
KEGG:04713	Circadian entrainment	7	7.2916665	4.34E-03	CAMK2A, CAMK2B, FOS, GNAO1, GRIN1, GRIN2B, MTNR1A
KEGG:04380	Osteoclast differentiation	8	6.060606	5.02E-03	AKT1, FOS, FYN, GRB2, IKBKG, JUN, STAT1, TRAF6
KEGG:05160	Hepatitis C	8	6.0150375	5.14E-03	AKT1, CDKN1A, EGFR, GRB2, IKBKG, STAT1, TP53, TRAF6
KEGG:04068	FoxO signaling pathway	8	5.970149	5.25E-03	AGAP2, AKT1, CDKN1A, EGFR, GABARAPL2, GRB2, MDM2, SMAD2
KEGG:04917	Prolactin signaling pathway	6	8.333333	5.77E-03	AKT1, ESR2, FOS, GRB2, SRC, STAT1
KEGG:05142	Chagas disease (American trypanosomiasis)	7	6.730769	6.15E-03	AKT1, FOS, GNAO1, IKBKG, JUN, SMAD2, TRAF6
KEGG:04660	T cell receptor signaling pathway	7	6.6666665	6.31E-03	AKT1, FOS, FYN, GRB2, IKBKG, JUN, NCK1
KEGG:04725	Cholinergic synapse	7	6.3063064	8.59E-03	AKT1, BCL2, CAMK2A, CAMK2B, FOS, FYN, GNAO1
KEGG:05213	Endometrial cancer	5	9.615385	9.08E-03	AKT1, EGFR, GRB2, MYC, TP53
KEGG:04724	Glutamatergic synapse	7	6.140351	9.39E-03	DLG4, DLGAP1, GNAO1, GRIN1, GRIN2B, SHANK1, SLC1A3
KEGG:04919	Thyroid hormone signaling pathway	7	5.9322033	1.11E-02	AKT1, MDM2, MYC, RXRB, SRC, STAT1, TP53
KEGG:05223	Non-small cell lung cancer	5	8.928572	1.14E-02	AKT1, EGFR, GRB2, RXRB, TP53
KEGG:04720	Long-term potentiation	5	7.4626865	2.51E-02	CAMK2A, CAMK2B, GRIN1, GRIN2B, PPP1CA
KEGG:04066	HIF-1 signaling pathway	6	5.8252425	2.68E-02	AKT1, BCL2, CAMK2A, CAMK2B, CDKN1A, EGFR
KEGG:04210	Apoptosis	7	5.0	2.71E-02	AKT1, BCL2, BID, FOS, IKBKG, JUN, TP53
KEGG:04620	Toll-like receptor signaling pathway	6	5.6603775	2.81E-02	AKT1, FOS, IKBKG, JUN, STAT1, TRAF6
KEGG:05218	Melanoma	5	7.0422535	2.83E-02	AKT1, CDKN1A, EGFR, MDM2, TP53
KEGG:04662	B cell receptor signaling pathway	5	6.849315	2.89E-02	AKT1, FOS, GRB2, IKBKG, JUN
KEGG:04520	Adherens junction	5	6.756757	2.90E-02	CSNK2B, EGFR, FYN, SMAD2, SRC
KEGG:05145	Toxoplasmosis	6	5.084746	3.83E-02	AKT1, BCL2, GNAO1, IKBKG, STAT1, TRAF6
KEGG:05030	Cocaine addiction	4	8.163265	4.04E-02	DLG4, GRIN1, GRIN2B, JUN

### BRI2 brain region specific interactome

**Figure 4:**
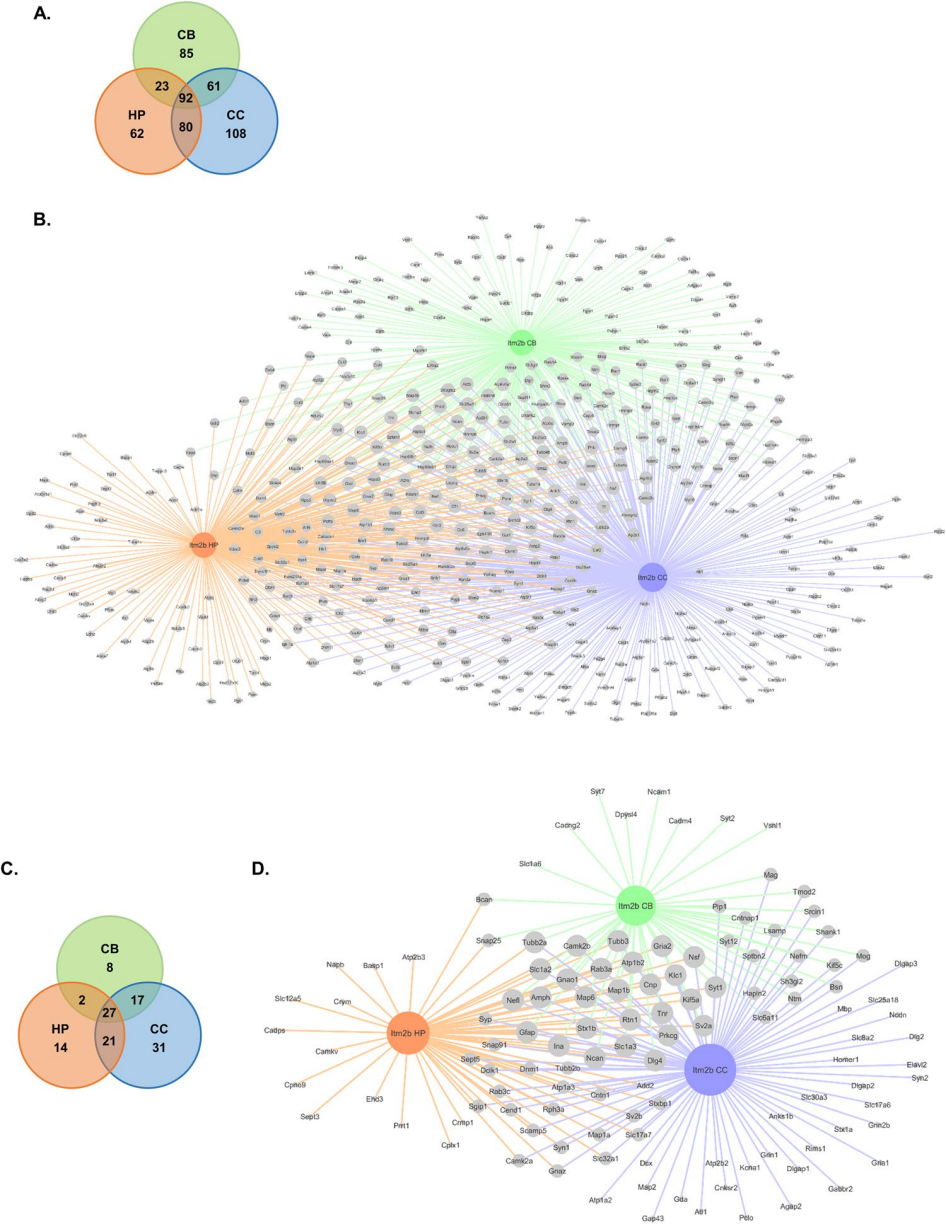
Brain region specific protein interactions in the BRI2 interactome. (**A**,**B**) Brain region specific protein interactions revealed in the identified BRI2 interactome. (**A**) Venn diagram comparison of the brain region (cerebral cortex, hippocampus, and cerebellum) specific BRI2 protein interactions identified in this study. (**B**) BRI2 interactome identified in this study. Node size according to the degree in the network. Edge color represents the source of interaction: blue edges correspond to the BRI2 interactions identified in the cerebral cortex, orange edges correspond to the BRI2 interactions identified in the hippocampus, and green edges correspond to the BRI2 interactions identified in the cerebellum. (**C**,**D**) Brain region specific protein interactions revealed in the identified BRI2 ES interactome. (**C**) Venn diagram comparison of the brain region (cerebral cortex, hippocampus, and cerebellum) specific BRI2 protein interactions identified in this study, highly enriched or specific for the brain. (**D**) BRI2 ES interactome identified in this study. Node size according to the degree in the network. Edge color represents the source of interaction: blue edges correspond to the BRI2 interactions identified in the cerebral cortex, orange edges correspond to the BRI2 interactions identified in the hippocampus, and green edges correspond to the BRI2 interactions identified in the cerebellum.

A second analytical approach used in this study of the BRI2 interactome, took into account the different brain regions, since this could be important and may reveal differences providing novel insights into specific BRI2 associated brain region-dependent processes. Therefore, the identification and characterization of the specific candidate BRI2 interactors from the three different brain regions: cerebral cortex, hippocampus, and cerebellum, was carried out (Fig. 4). A comparative analysis of the 511 candidate BRI2 interacting proteins identified in this study revealed that 108 proteins were specifically identified in the cerebral cortex, 62 in the hippocampus and 85 in the cerebellum (Fig. 4A). Further, 92 proteins were common to all regions, while 61 were shared by the cerebral cortex and cerebellum, 23 between cerebellum and hippocampus and 80 between cerebral cortex and hippocampus (Fig. 4A and B). Furthermore, given the particular interest in studying BRI2 function(s) in the CNS (central nervous system), a comparative analysis of the BRI2 ES interactome was also carried out (Fig. 4C). The results evidenced that while the majority of proteins were shared between brain regions (27 shared in all three regions, 17 shared in the cerebral cortex and cerebellum, 21 shared in cerebral cortex and hippocampus, and 2 shared in cerebellum and hippocampus), a subset of proteins were found to co-IP with BRI2 in specific brain regions (31 in cerebral cortex, 14 in hippocampus, and 8 in cerebellum) (Fig. 4C).

Once the BRI2 ES region specific interactomes were established (Fig. 4D), network augmentation was performed to enhance the experimental data with additional known and experimentally validated protein partners and obtain PPI networks (BRI2 ES region-specific networks). For the cerebral cortex specific PPI network (Supplementary Figure S2A), a total of 292 proteins (nodes) with 330 connections (edges) between them was mapped. This network presents an average number of 2.243 neighbors. Regarding the hippocampus specific PPI network (Supplementary Figure S2B), 160 proteins, with 165 connections between them was mapped. The topological analysis revealed an average number of neighbors of 2.0625. The cerebellum-specific PPI network (Supplementary Figure S2C) was the smallest, with only 47 nodes connected by 47 edges, and with an average of 2.0 neighbors. To further address if the BRI2 interactome could be assigned to selective brain region-dependent processes, functional enrichment analysis of the BRI2 ES region-specific networks, using the PANTHER online resource, was performed (Table 3). Remarkably, biological process annotation evidenced that for the three BRI2 ES region-specific networks, the highly enriched terms were similar to those previously associated with the BRI2 ES interactome, namely neuron differentiation and synaptic vesicle cycling (Table 3 and Fig. 2A).

**Table 3:** Biological process enrichment analysis of the BRI2 ES regional specific networks (cerebral cortex, hippocampus, and cerebellum) using Panther online resource. Enriched categories are identified as those with p-value < 0.05, and GO terms presented correspond to the two most specific for each category retrieved in the analysis. Nr. Number.

GO term	Nr. proteins	Fold enrichment (%)	p-value	Associated proteins
**BRI2 ES cerebral cortex specific network analysis**				
synaptic vesicle fusion to presynaptic active zone membrane (GO:0031629)	9	55.78	1.28E-09	SNAP25, SNAP23, SNAP47, STX3, STX2, SNAP29, STX11, STX1A, STX4
vesicle fusion with Golgi apparatus (GO:0048280)	6	44.62	5.70E-05	VTI1B, SEC. 22B, BET1, GOSR2, STX5, SEC. 22 A
dendrite morphogenesis (GO:0048813)	9	13.66	2.62E-04	DLG4, ITGB1, FYN, DCX, ABI1, SHANK3, SHANK1, CAMK2A, MAP2
protein localization to membrane (GO:0072657)	22	4.07	3.48E-04	RTP2, TNF, VAMP4, VAMP3, VAMP2, DLG4, SNAP25, ITGB7, SNAP47, NUP54, DLG2, TMED2, STX3, SHANK3, DLG1, DLG3, STX1A, NSF, VAMP5, EGFR, RPS8, TNIK
learning (GO:0007612)	13	7.11	5.05E-04	DLG4, SNAP25, SLC8A2, ITGB1, SHANK2, FYN, HTT, SHANK3, ATP1A2, SHANK1, APP, PARK2, GRIN1
regulation of protein localization to plasma membrane (GO:1903076)	10	10.47	5.25E-04	VTI1B, ITGB1, PIK3R1, STX8, STX3, GOPC, DLG1, STX7, EGFR, STX4
ERBB2 signaling pathway (GO:0038128)	8	15.26	6.70E-04	GRB2, CDC37, PTPN12, SRC, PIK3R1, ERBB4, ERBB2, EGFR
long-term synaptic potentiation (GO:0060291)	8	14.87	8.11E-04	VAMP2, SNAP25, SLC8A2, SHANK2, SNAP47, STX3, SHANK3, STX4
retrograde vesicle-mediated transport, Golgi to ER (GO:0006890)	10	9.18	1.74E-03	TMED9, RINT1, SEC. 22B, KDELR1, TMED2, KIF18A, HTT, USE1, BNIP1, NSF
retrograde transport, endosome to Golgi (GO:0042147)	10	9.07	1.95E-03	TRIM27, VAMP3, TMED9, DCTN1, VTI1B, STX10, STX16, GOSR2, STX5, STX6
post-Golgi vesicle-mediated transport (GO:0006892)	10	8.55	3.32E-03	VAMP4, VAMP3, VAMP2, VTI1B, SNAP23, GOSR2, GOPC, NSF, VAMP5, STX4
receptor localization to synapse (GO:0097120)	5	33.80	4.14E-03	DLG4, DLG2, ANKS1B, DLG1, DLG3
regulation of ion transport (GO:0043269)	25	3.17	4.50E-03	TRIM27, CREB3, VAMP3, KCNA3, PRKACA, VAMP2, PLN, DLG4, GPM6B, YWHAE, KCNA1, PLCG1, HTT, SHANK3, ATP1A2, PRKCD, ATP2B2, ABL1, HOMER1, SHANK1, DLG1, CALCA, KCNA10, CAMK2A, PARK2
vesicle-mediated transport to the plasma membrane (GO:0098876)	9	9.70	4.52E-03	VAMP4, VAMP3, VAMP2, SNAP25, SNAP47, STX3, GOPC, NSF, VAMP5
positive regulation of excitatory postsynaptic potential (GO:2000463)	6	20.28	5.65E-03	RIMS1, DLG4, SHANK3, SHANK1, STX1A, GRIN1
GDP metabolic process (GO:0046710)	5	28.60	9.33E-03	MAGI3, DLG4, DLG2, DLG1, DLG3
neuromuscular process (GO:0050905)	10	7.36	1.25E-02	DLG4, KCNA1, PTPRQ, SHANK3, ATP2B2, ABL1, SHANK1, APP, PARK2, GRIN1
SNARE complex assembly (GO:0035493)	4	49.58	1.33E-02	VAMP4, VAMP3, VAMP1, STX4
protein localization to synapse (GO:0035418)	6	17.16	1.47E-02	DLG4, SNAP25, SNAP47, STX3, SHANK1, PCLO
regulation of intracellular transport (GO:0032386)	22	3.24	1.54E-02	CREB3, TNF, VAMP3, RIMS1, VAMP2, PLN, NAPB, SRC, EMD, YWHAE, RINT1, NUP54, PIK3R1, RNASE2, ERBB4, ERBB2, PRKCD, HMOX1, MDM2, STX1A, PARK2, EGFR
peptidyl-tyrosine phosphorylation (GO:0018108)	13	5.14	1.83E-02	TRIM27, INSR, CDC37, SRC, ABI3, FYN, PIBF1, ERBB4, ERBB2, ABI1, PRKCD, ABL1, EGFR
COPII vesicle coating (GO:0048208)	8	9.75	1.86E-02	GOLGA2, GRIA1, SEC. 22B, BET1, TMED2, GOSR2, STX5, NSF
activation of protein kinase activity (GO:0032147)	16	4.09	2.38E-02	TNF, AGAP2, INSR, ITGB3, PRKACA, SRC, PIBF1, CRK, PLCG1, PRKCD, ABL1, CD81, DLG1, CALCA, EGFR, TNIK
synaptic vesicle docking (GO:0016081)	4	37.18	4.12E-02	SNAP25, STX3, BLOC1S6, STX1A
**BRI2 ES hippocampus specific network analysis**				
synaptic vesicle fusion to presynaptic active zone membrane (GO:0031629)	7	77.92	6.23E-08	STX19, STX1B, STX3, STX2, SNAP29, STX1A, STX4
cytoskeleton organization (GO:0007010)	28	3.97	3.85E-06	CRMP1, PFN1, DPYSL3, UBE2B, MYO18A, KLHL20, DPYSL2, ACTR2, SRC, FLNA, ADD2, DBN1, SDCBP, LIMA1, ZAK, ANK2, KIF18A, HTT, PIP5K1A, ANLN, SPRY2, SEPT1, SYNE4, CCDC155, AXIN1, MYH9, VIM, CDK5RAP2
vesicle docking (GO:0048278)	9	20.73	6.79E-06	STX19, STX1B, STX3, STX2, STX16, STX5, STX1A, STX4, STX6
intracellular protein transport (GO:0006886)	23	4.15	7.66E-05	AP3M1, CDC37, NAPB, STX19, EHD3, YWHAE, SDCBP, STX1B, VCP, STX3, MICALL1, ANK2, STX2, PIP5K1A, MYO1C, MCM3AP, STX16, BID, STX5, ARL6IP1, STX1A, STX4, STX6
cell projection organization (GO:0030030)	25	3.23	1.95E-03	CRMP1, MAPK8IP2, UBE2B, IQCB1, RTN4, DPYSL2, HMGB1, ACTR2, SRC, FLNA, EHD3, TMEM17, YWHAE, SDCBP, RPS6KA5, LIMA1, INTU, STX3, MICALL1, IQGAP1, PIP5K1A, SNAP29, SLC12A5, MYH9, CDK5RAP2
Golgi vesicle transport (GO:0048193)	14	5.62	2.22E-03	MYO18A, KLHL20, EHD3, RBSN, BET1, TFG, VCP, ANK2, KIF18A, HTT, STX5, STX4, ANKFY1, STX6
spindle localization (GO:0051653)	6	20.04	5.83E-03	ACTR2, HTT, SPRYZ, CCDC155, MYH9, CDK5RAP2
cell differentiation (GO:0030154)	50	1.95	1.00E-02	CRMP1, DPYSL3, MAPK8IP2, UBE2B, MAPK6, RTN4, ATF2, DPYSL2, HMGB1, ACTR2, SRC, FLNA, AGR2, DBN1, IL33, YWHAE, PSMD11, RPS6KA5, BASP1, STX1B, INTU, STX3, CDK5RAP3, ZAK, MICALL1, ANK2, IQGAP1, STX2, RNF8, FXR1, PIP5K1A, ANLN, SMPD3, SPRY2, HNRNPH3, RIPK3, SYNE4, SLC12A5, HSPE1, CCDC155, AXIN1, GNB2L1, MYH9, ANXA7, VIM, TRIP13, CDK5RAP2, CPNE9, FAS, RGS2
protein complex assembly (GO:0006461)	23	3.04	1.75E-02	DPYSL3, MAPK8IP2, TK1, RTN4, SRC, ADD2, EHD3, PSMD11, BET1, TFG, VCP, CADPS, NDUFV2, STX2, AMFR, ANLN, SNAP29, RIPK3, BID, STX5, AXIN1, STX4, FAZ
regulation of cysteine-type endopeptidase activity involved in apoptotic process (GO:0043281)	10	6.61	2.92E-02	HMGB1, SRC, YWHAE, VCP, SERPINB9, BID, ARL6IP1, HSPE1, GNB2L1, FAZ
actin filament-based process (GO:0030029)	15	4.14	3.50E-02	PFN1, DPYSL3, MYO18A, ACTR2, SRC, FLNA, ADD2, DBN1, SDCBP, LIMA1, ANK2, PIP5K1A, CCDC155, MYH9, VIM
supramolecular fiber organization (GO:0097435)	13	4.72	3.97E-02	DPYSL3, ACTR2, SRC, FLNA, ADD2, DBN1, LIMA1, KIF18A, RIPK3, CCDC155, AXIN1, VIM, HSP90AB1
positive regulation of intracellular signal transduction (GO:1902533)	21	3.08	4.21E-02	MAPK8IP2, RGL2, PELI2, HMGB1, SRC, FLNA, SDCBP, TFG, CDK5RAP3, ZAK, IQGAP1, HTT, PLA2G2A, SPRY2, RIPK3, BID, CCL18, AXIN1, GNB2L1, EEF1D, FAS
cytosolic transport (GO:0016482)	8	8.62	4.29E-02	KLHL20, ACTR2, EHD3, RBSN, STX16, STX5, ANKFY1, STX6
**BRI2 ES cerebellum-specific network analysis**				
synaptic vesicle recycling (GO:0036465)	4	60.13	5.56E-03	SH3GL2, SYT2, SYT7, SH3GL1
endocytosis (GO:0006897)	9	7.44	2.22E-02	SH3GL3, SH3GL2, WIPF1, DPYSL2, SYT2, SYT7, CSNK1E, SH3GL1, CACNG2
developmental process (GO:0032502)	28	2.42	2.29E-03	SH3GL3, COL10A1, SH3GL2, GFI1B, DPYSL4, DPYSL2, PRKD2, RHEB, SYT2, SIAH1, ITM2B, KIF18A, PTPRO, HTT, SRPK2, CSNK1E, SH3GL1, SPRED1, TFF1, LITAF, SYNE4, UCHL1, CCDC155, TRIP13, ARIH2, SPRY3, SPRED2, NCAM1

### Validation of HPLC-MS/MS-identified BRI2 interactors by immunoblotting analysis

**Figure 5:**
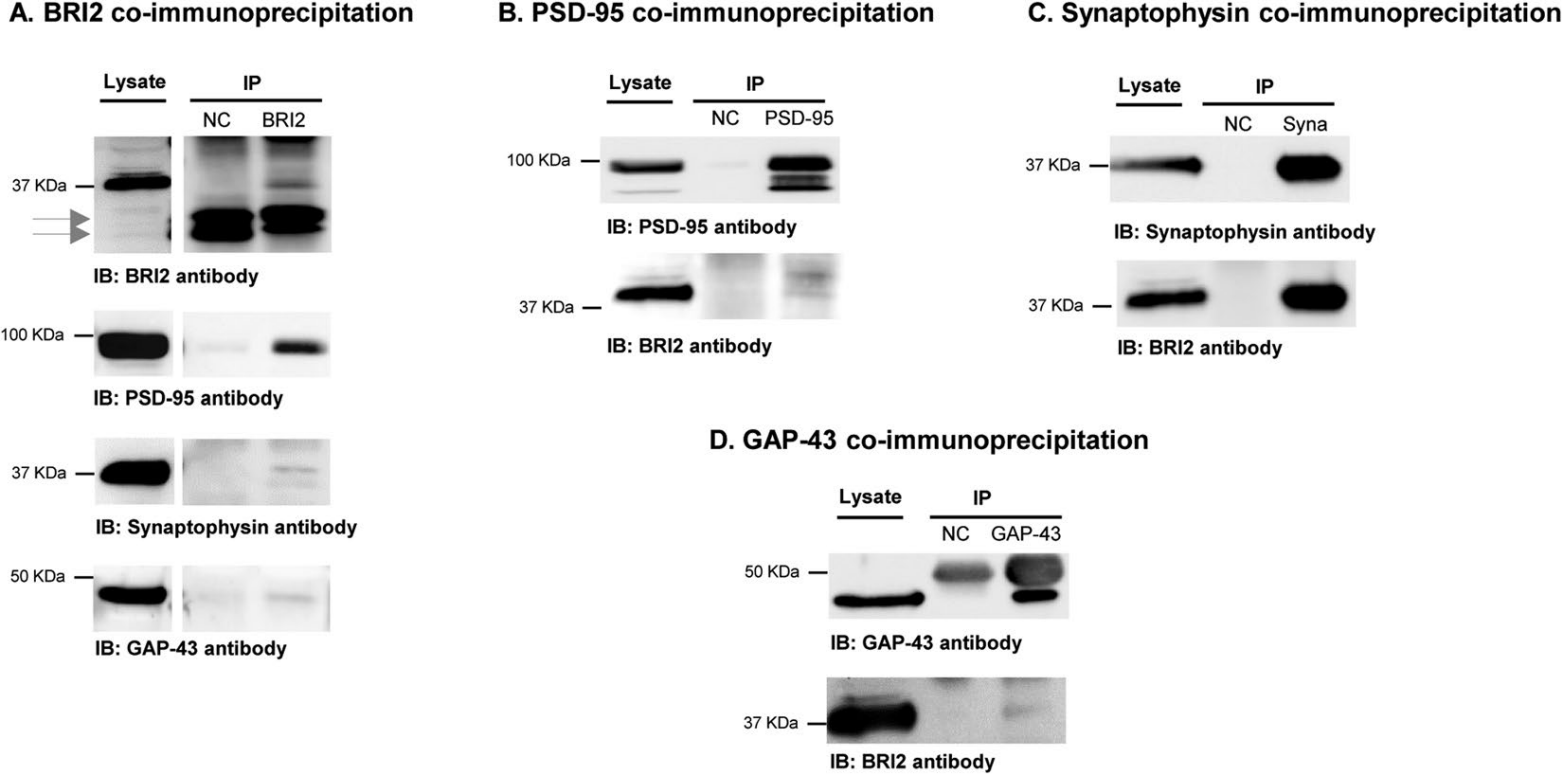
Co-immunoprecipitation of BRI2, PSD-95, synaptophysin and GAP-43 in rat cerebral cortex lysate. Rat cerebral cortex lysate was immunoprecipitated with a mouse monoclonal BRI2 antibody (**A**), a mouse monoclonal PSD-95 antibody (**B**), a mouse monoclonal synaptophysin antibody (**C**) and a rabbit polyclonal GAP-43 antibody (**D**) bound to protein G dynabeads. The mock IP negative controls were performed by incubating cell extracts with G Dynabeads coupled with mouse IgG or rabbit IgG. NC, mock IP negative controls. Arrows indicate BRI2 N-terminal fragments detected in brain lysates.

The identification of DLG4, also known as PSD-95, synaptophysin (SYP), and GAP-43 in the BRI2 ES interactome is of paramount importance since it suggests the presence of BRI2 in both the pre- and postsynaptic compartments, and in growth cones. For this reason, PSD-95, synaptophysin and GAP-43 were selected for additional biochemical validation (i.e., immunoblotting). Briefly, co-IPs were carried out for BRI2, PSD-95, synaptophysin and GAP-43 from the cerebral cortex using a specific antibodies, and the respective mock negative controls. Analyses were carried out by SDS-PAGE and immunoblotting (Fig. 5). First, it was possible to confirm that the BRI2 co-immunoprecipitation approach was effective, as when a BRI2 antibody was used in immunoblotting, a signal in the range of 37–40 kDa was detected, corresponding to the full-length BRI2 protein (Fig. 5A). Complexing with each of mentioned proteins was confirmed given that by using the PSD-95, synaptophysin and GAP-43 specific antibodies, a positive signal for each of these interactors was obtained. In each case the BRI2 co-IP are consistent with the molecular weights; PSD-95 (95–100 KDa), synaptophysin (37 KDa) and GAP-43 (43 KDa) (Fig. 5A). The reverse co-immunoprecipitations were also effective, since specific PSD-95 (Fig. 5B), synaptophysin (Fig. 5C) and GAP-43 (Fig. 5D) antibodies co-IPed full-length BRI2. Therefore, these results validate the mass spectrometry data, corroborating the formation of a three novel containing BRI2 complexes with PSD-95, synaptophysin and GAP-43 (Fig. 3 and Table 1).

## Discussion

BRI2 is a relevant protein in the pathogenesis of the neurodegenerative diseases FBD and FDD, as well as in AD, given its association with APP and its effects on Abeta production, aggregation and degradation^7,8,20,21^. Several lines of evidence suggest a role for BRI2 at the nerve terminals^4^ and in neurite outgrowth^3,5,6^, however, its precise physiological function and associated signalling pathways remain elusive. The study of PPI networks is a valuable methodology to uncover the role of previously uncharacterized proteins in pathways or protein complexes, both in healthy and diseased states. Therefore, the identification of the BRI2 interactome is crucial for understanding the biological functions of BRI2 in the CNS. This study focused on elucidating and characterizing the BRI2 brain interactome, which was isolated from rat cerebral cortex, hippocampus and cerebellum by co-immunoprecipitation assays, using a specific BRI2 antibody. This approach identified 511 protein candidates as potential BRI2 brain interactors, of which two were previously reported in public protein interaction databases: Ppp1ca and Cacna2d1. PP1 is a major serine/threonine phosphatase that was recently found to interact directly with BRI2. The BRI2:PP1 complex seems to be relevant in the nervous system since BRI2 dephosphorylation by PP1 appears to be responsible for both the regulation of BRI2 processing and its role in neurite outgrowth^5,6^. In total, only two of the previously reported BRI2 interactors (Supplementary Table S3) were identified in the work here presented. Of note, only a small number of these previously reported BRI2 interacting proteins have actually been validated. Moreover, the failure to identify other known BRI2 interactors might be due to several factors. For instance, limitations of this study include the possibility of poor recovery of the membrane and nuclear proteins, not detecting weak or transient interactions, and the cellular/tissue expression of the proteins previously detected. It is also crucial to take into account that the HPLC-MS/MS technique identifies co-complex interactions which include direct physical interactions but also indirect co-complex associations^22^. Remarkably, although the BRI2 interactors here identified do not present a high overlap with the others previously reported in the literature, many were functionally similar or related. It is also important to note that a specific BRI2 antibody that recognizes the N-terminal of the protein was used. Since BRI2 suffers proteolytic cleavages that originate different BRI2 N-terminal fragments, it is possible that the co-IP experiments result in both the full-length BRI2 and the different BRI2 N-terminal fragments.

In this study, the BRI2 brain interactome was identified, and thus a tissue-specific network-based approach was used. Of the 511 candidate BRI2 brain interactors identified in this study, 120 were highly specific or strongly expressed in the brain. Functional enrichment analysis for cellular component of the BRI2 ES interactome suggests a subcellular localization for the BRI2 protein in neuron projections, synapses, both presynapse, and postsynapse, and associated with synaptic vesicles (Fig. 2B). These results together with the fact that the majority of these BRI2 interactors are cytoskeletal proteins, transporters and membrane traffic proteins (Fig. 1) are consistent with the concept that BRI2 in the CNS has a role in nerve terminals and neuronal differentiation^3–6^. Analysis of the BRI2 ES interactome resulted in the identification of proteins associated with several processes of the synaptic vesicle cycle, such as neurotransmitter uptake and vesicle docking, priming, fusion and exocytosis (Fig. 2A and Supplementary Table S5).

Moreover, several proteins in the BRI2 ES interactome associated with the microtubule transport machinery were found, in particular, β-tubulin isotype III (Tubb3b), β-tubulin isotype IIb (Tubb2b), kinesin heavy chain isoforms 5a and 5c (Kif5a and kif5c, respectively), and kinesin light chain isoform 1 (Klc1). Therefore, these results suggest that in neurons, BRI2 is transported within axons by anterograde axonal transport to the distal nerve terminals where the full-length protein or even its N-terminal fragments may interact with the neurotransmitter machinery release and contribute to synaptic signalling.

The present results regarding the BRI2 ES interactome also suggest an association for BRI2 with synaptic transmission, plasticity, and learning. Several BRI2 brain interactome members including disk large homolog 4 (Dlg4/PSD-95), calcium/calmodulin-dependent protein kinase II alpha (Camk2a/CamkIIα), calcium/calmodulin-dependent protein kinase II beta (Camk2b/CamkIIβ), protein phosphatase 1 (PP1/Ppp1ca), (glutamate receptor ionotropic NMDA1 (Grin1/NR1/GluN1), glutamate receptor ionotropic NMDA2B (Grin2b/GluN2B/NR2B), glutamate receptor ionotropic AMPA1 (Gria1/GluA1/GluR-1) and glutamate receptor ionotropic AMPA2 (Gria2/GluA2/GluR-2) participate in these processes. The PSD-95 protein was identified in our interactome firstly by MS analysis (Fig. 3) and further validated by immunoblotting analysis as novel BRI2 interactor (Fig. 5), suggesting the presence of BRI2 in the postsynaptic compartments. One previous study by using gene-targeted affinity purification in mice (TAP-tagged PSD-95 knockin mice) followed by mass-spectrometry identification allowed the identification of several known and new putative PSD-95 interactors in mice brain extracts, and BRI2 was not identified^23^. However, we should mention that routinely affinity purification coupled to mass spectrometry protocols recovers only the most stable interactions, and the transient biological interactions in nature that depends on the cellular environment can be missed.

PSD-95 is a major scaffold protein in the brain and concentrated in the postsynaptic density (PSD) of excitatory synapses, implicated in synapse maturation and in regulating synaptic strength and plasticity^24–26^. PSD-95 interacts with both AMPA receptors (AMPARs) and NMDA-type glutamate receptors (NMDARs). Therefore, it is plausible to hypothesize that BRI2 may also be involved in synaptic transmission and plasticity by altering the AMPAR and NMDAR trafficking which affects excitatory synaptic transmission. Consistent with a role for BRI2 in synaptic plasticity, learning and memory, *Itm2b* (+/−) mice exhibit synaptic and memory deficits. Knock-in mouse models of FDD and FBD, FDD_KI_ and FBD_KI_ respectively, exhibit similar deficits which are attributed to the loss of BRI2 function in those neurodegenerative diseases^27,28^. Moreover, in a synaptosomal proteome characterization of the mouse model FDD_KI_, PSD-95 expression was found decreased and pointed as a potential player in the altered synaptic transmission and activity observed in this mice^29^. PSD-95 knockout mice display several phenotypic similarities, such as abnormal synaptic plasticity as well as abnormal memory and learning^30^.

An exciting feature of these BRI2 interactomes is the high number of cytoskeleton proteins which translated into an overrepresentation of functions like cytoskeleton organization and microtubule-based processes, evidently important for neuronal differentiation (Fig. 2A). Rearrangements of actin and microtubules cytoskeletons are crucial to establish and maintain polarity in neurons, in neural migration and vesicle trafficking^31,32^. In fact, almost 50% (49 out of the 120) of the brain BRI2 ES interactome proteins are annotated with neuronal differentiation related biological processes (Supplementary Table S7), and the construction of a BRI2-neuronal differentiation-specific PPI subnetwork (Fig. 3) allowed us to explore the biological functionalities and the potential underlying molecular mechanisms of BRI2 in these processes. In line with this observation, functional enrichment pathway analysis of the expanded BRI2-neuronal differentiation-specific interactome associated BRI2 interactors to signalling pathways involved in several neuronal differentiation related events, namely adherens junction, axon guidance, ErbB signalling pathway^33,34^, FoxO signalling pathway^35^, neurotrophin signalling pathway^36^ and estrogen signalling pathway^37^ (Table 2). These results strengthen the proposed role for BRI2 in neuronal differentiation and neurite outgrowth and an association of BRI2 with these signalling pathways deserve further investigations.

The identification of adhesion proteins, such as neural cell adhesion molecule (Ncam1), a member of the Ig superfamily of cell-adhesion molecules^38^ in the study here presented leads us to propose that one of the mechanisms by which BRI2 promotes neurite outgrowth might be by regulating cell-cell adhesion. Ncam associates with numerous cytoskeletal components but also with some signalling pathways associated with a neurite outgrowth response^39^. For instance, Ncam interacts with spectrin, a protein involved in linking cellular membranes to the actin filament cytoskeletal system, which in turns interacts with the growth-associated protein 43 (GAP-43), a protein that is highly expressed in growth cones and believed to be important for growth cone extension. Likewise, Ncam-mediated neurite outgrowth was inhibited in neurons from GAP-43 knockout mice, supporting a functional relationship between Ncam and GAP-43^39^. Remarkably, both spectrin (Sptbn2) and GAP-43 (Gap43) were identified in our BRI2 interactome and therefore these mechanisms deserve further investigation. Moreover, it has already been described that BRI2 is able to form homodimers linked by disulfide bonds that appear at the cell surface resembling the structure of a cell-surface receptor^40^. Although a receptor function for BRI2 has not been established it is possible to hypothesize that BRI2, triggered by ligand interaction, may act as a cell surface receptor participating in the above-mentioned signalling pathways. It was recently demonstrated by our group that BRI2 phosphorylation modulates its proteolytic processing and neuritogenic role. Moreover, for BRI2 mediated neurite outgrowth, it appears that phosphorylation of full-length protein promotes the emergence of neurites whereas the increased BRI2 NTFs play a relevant role in neurites’ elongation and stabilization^6^. Thus, it is also plausible to assume that mechanisms underlying BRI2 role in neurite outgrowth may also involve: (1) BRI2 full-length phosphorylation induces conformational changes in the protein which affect protein-protein binding, and for instance could affect binding to neurotrophic effectors of the above signalling pathways; (2) increased BRI2 proteolytic processing to its secreted fragment (containing the BRICHOS domain) which can activate the above signalling pathways via a growth factor receptor.

Finally, a comparative analysis approach of the BRI2 ES interactome in the three different brain regions was employed, resulting in the identification of 31 proteins specifically in the cerebral cortex, 14 in the hippocampus, and 8 in the cerebellum (Fig. 4C). The results evidenced that in the different brain regions BRI2 seems to be associated with cellular processes also highlighted in the analysis of the total BRI2 ES interactome, namely neuron development and synaptic vesicle cycle (Table 3), supporting that the majority of the biological functions of BRI2 in the CNS are in fact relatively stable across the three brain regions.

In summary, these results provide important insights on BRI2 brain interactome, comprising several new putative interacting partners. We observed a complex interaction of BRI2 with proteins related to neuronal differentiation, neurite outgrowth, synaptic signalling and plasticity. Further, we validated PSD-95, synaptophysin and GAP-43 as novel BRI2 interactors in the brain using co-IP and immunoblotting analysis. Therefore, the work here presented provides a valuable list of BRI2 interacting proteins for further studies to investigate their potential roles in BRI2 biology and the underlying molecular mechanisms, as well as novel insights in its associated pathologies (FBD, FDD, and AD).

## Methods

### Preparation of rat brain lysates

Wistar Hannover rats (9–12 weeks) were obtained from Harlan Interfaune Ibérica, SL, and all experimental procedures were conducted in accordance with the European legislation for animal experimentation (2010/63/EU). No specific ethics approval under EU guidelines was required for this project, since the rats were euthanized, by cervical stretching followed by decapitation, for brain removal. This is within the European law (Council Directive 86/609/EEC) and the number and suffering of the animals were minimized as much as possible. The procedures were approved and supervised by our Institutional Animal Care and Use Committee (IACUC): Comissão Responsável pela Experimentação e Bem-Estar Animal (CREBEA). Briefly, animals were sacrificed by cervical stretching followed by decapitation and the cerebral cortex, hippocampus and cerebellum were dissected on ice. Tissues were further weighed and homogenized on ice with a Potter-Elvehjem tissue homogenizer with 10–15 pulses at 650–750 rpm, in non-denaturating lysis buffer (50 mM Tris-HCl pH 8.0, 120 mM NaCl and 4% CHAPS) containing protease inhibitors (1 mM PMSF, 10 mM Benzamidine, 2 µM Leupeptin, 1.5 µM Aprotinin and 5 µM Pepstatin A)^41^. The tissue extracts were used for immunoprecipitation analysis as described below.

### Co-immunoprecipitation

The lysates from rat cerebral cortex, hippocampus and cerebellum extracts were divided into three equal parts (2 controls and 1 BRI2 co-immunoprecipitation) and pre-cleared separately using Dynabeads Protein G (Life Technologies). A direct immunoprecipitation approach was performed using mouse anti-BRI2 antibody (1 µg/500 µg protein; Cat. No. sc-374362, Santa Cruz Biotechnology), pre-incubated with Dynabeads Protein G for 1 hour at 4 °C with rotation. After, the pre-cleared extracts were applied to antibody-Dynabeads and incubated overnight at 4 °C with rotation. The immunoprecipitates were washed three times with PBS in 3% BSA for 10 min at 4 °C with rotation and beads were re-suspended in 1% SDS^42^. As a control extracts only incubated with dynabeads protein G or with dynabeads protein G coupled with mouse IgGs were used. Each brain region received the whole procedure three independent times.

### In-gel protein digestion, nano-HPLC and mass spectrometry

The eluted fractions from the brain BRI2 co-IP assays were separated by SDS-PAGE on a 12% Bis-Tris gel and gels bands excised, cut into small pieces, and alternately washed with buffer A (50 mM ammonium hydrogen carbonate (NH4HCO3)) and B (50 mM NH4HCO3/50% ACN (acetonitrile)). Following dehydration of gel pieces *in vacuo*, trypsin (Serva, Germany) was solved in 10 mM HCl and 50 mM NH4HCO3 and used for overnight in gel digestion at 37 °C (trypsin:protein ratio 1:20). Peptides were then extracted once with 200 μl of 50% ACN/0.05% TFA (trifluoroacetic acid) and once with 100 μl of 50% ACN/0.05% TFA. Extracts were combined and centrifuged for 5 min at 16,000 × g. The supernatant was transferred to a new vial in order to get rid of potential gel residuals. Then, ACN was removed *in vacuo*. For LC–MS analysis, a final volume of 16 μl was prepared by addition of 0.1% TFA.

Peptide extracts were used for mass spectrometry analysis. Therefore, samples were injected via the autosampler of an RSLC nano system (Thermo Scientific), concentrated on a C_18_ trapping column (2 cm length, 100 μm i.d., 5 μm particle size, Thermo Scientific), and separated on a C_18_ analytical column (50 cm length, 75 μm i.d., 2 μm particle size, Thermo Scientific) heated at 60 °C before being emitted via a coated silica tip (FS360-20-10-D-20, New Objective, USA) of the nanospray-electrospray source of an Q Exactive (Thermo Scientific). The HPLC separation was performed with a gradient method of in total 120 min consisting of: 7 min of loading the sample and washing the column with 0.1% TFA at a flow rate of 30 μl min^−1^ on the trapping column, followed by separation applying a linear gradient at a flow rate of 400 nl min^−1^ with the solvents A (0.1% FA (formic acid) in HPLC grade water) and B (84% ACN/0.1% FA in HPLC grade water) starting from 5% B to 40% B in 98 min on the heated analytical column, a linear gradient of 40% B to 95% B in 2 min, and washing for 7 min with 95% B. Finally, a gradient was applied from 95% B to 5% B in 1 min followed by equilibration for 5 min with 5% B. For ionization a spray voltage of 1.6 kV and capillary temperature of 250 °C was used. The acquisition method consisted of two scan events, Full MS and MS/MS. The Full MS was monitored from m/z 350 to 1400, with an Orbitrap resolution of 70,000, a maximum injection time of 80 ms and an automatic gain control (AGC) value of 3e6. The m/z values initiating MS/MS were set on a dynamic exclusion list for 35 s. Lock mass polydimethylcyclosiloxane (m/z 445.120) was used for internal recalibration. The 10 most intensive ions (charge N1) were selected for MS/MS-fragmentation and fragments were scanned at a resolution of 35,000, with a maximum injection time of 120 ms and an AGC value of 2e5. Fragments were generated by higher-energy collisional-induced dissociation (HCD) on isolated ions.

For peptide identification.raw files were processed in Proteome Discoverer 1.4 and analyzed using the Mascot search algorithm with a mass tolerance of 10 ppm and a fragment mass tolerance of 0.02 Da. Searches were performed allowing one missed cleavage site after tryptic digestion. Oxidation (M) and phosphorylation (STY) were considered as variable modifications. Targeted Decoy PSM Validator was implemented with an FDR of 1%. All data were searched against a database containing the whole Uniprot/Swissprot entries of the taxonomy *Rattus norvegicus*.

For each sample condition (i.e. for each specific brain region), two mock IPs were used as negative controls. Therefore, the proteins identified in the mock IPs were excluded from the list of the proteins identified in the BRI2 co-IPs, with one exception: the ratio between the number of peptide spectrum matches (PSM) for a given protein in a BRI2 co-IP sample and the PSM in the mock IPs, of the same brain region, were equal or higher than 2.0. In addition, well-described common contaminants as presented in^43^ and proteins characteristic of hair, skin/epidermis, tongue and gums (KRT33A, KRT78, KRT85, KRT14, KRT5, KRT3, KRT31, KRT32, KRT33B, KRT35, KRT36, KRT4, KRT77, KRT8, KRT17) which are also highly probable contaminants, were excluded.

### Bioinformatic data analysis

Experimentally detected BRI2 protein-protein interactions already described in the literature were retrieved using PSIQUIC View web service^44^ (version 1.4.5, downloaded October 2016), which enabled access to multiple PSI-MI compliant resources. In addition, two BRI2 interacting proteins identified and validated recently in our laboratory, protein phosphatase 1 alpha (PPP1CA) and protein phosphatase 1 gamma (PPP1CC), were added manually.

Tissue expression analysis was performed using the following databases: The Human Protein Atlas (HPA; available from www.proteinatlas.org)^45^; TiGER (available from http://bioinfo.wilmer.jhu.edu/tiger)^46^; and UniGene^47^. The HPA was searched for proteins strongly expressed in the brain and a list of proteins within the brain ‘Tissue enriched’, ‘Tissue enhanced’ and ‘Group enriched’ categories was obtained. Annotated protein expression is a manually curated score based on IHC staining patterns in normal tissues from two or more paired antibodies binding to different epitopes of the same protein, which describes the distribution and strength of expression of each protein in cells. The TiGER database was searched for proteins, preferentially expressed in the brain, based on ESTs by searching ‘Brain’ in ‘Tissue View’. The UniGene database was searched for brain-restricted genes by using the following search criteria: [brain][restricted].

Functional enrichment analysis of Gene Ontology (GO) categories (biological process and cellular component) was performed using PANTHER (Protein Annotation Through Evolutionary Relationship; version 11.1, accessed January 2016), online resource^48^. For the PANTHER enrichment analysis, the overall set of rat/human protein-coding genes were used as a reference set, and only enriched annotation with a p-value < 0.05 were considered. In addition, functional enrichment analysis of KEGG pathways was performed using the ClueGo plugin version 2.3.2^19^, (accessed January 2016) of the Cytoscape software version 3.4.0, freely available online^15^. Construction of protein-protein interaction (PPI) networks was achieved also using the Cytoscape software and further analyzed using the NetworkAnalyzer plugin version 3.3.1 (accessed January 2016). In order to perform network augmentation, protein-protein data was retrieved from the International Molecular Exchange (IMEx) consortium partners. Given the lack of information for *Rattus norvegicus* in these databases, the lists of proteins were extrapolated by homology to *Homo sapiens*. Rat UniProt accession numbers were converted to human using the Uniprot tool available online - Uniprot Retrieve/ID mapping (http://www.uniprot.org/uploadlists/)^49–66^.

### Interaction validation by immunoblotting

In order to validate BRI2 interactions, immunobloting assays were carried out. Brain lysates and eluted proteins from the co-immunoprecipitation assays performed in rat cortex tissue were resolved on 5–20% gradient SDS-PAGE and electrophoretically transferred onto nitrocellulose membranes. Moreover, additional co-immunoprecipitations were performed as described above, using a mouse anti-PSD-95 antibody (1 µg/100 µg protein; Cat. No. MAB1598; Millipore), a mouse anti-synaptophysin (5 µg/IP; Cat. No. 101011; Synaptic Systems) and a rabbit anti-GAP-43 antibody (1 µg/100 µg protein; Cat. No. 442695; Calbiochem).

Nonspecific protein-binding sites were blocked using 5% nonfat dry milk in TBST. Afterward, the membranes were incubated with the following primary antibodies: anti-BRI2 antibody (catalog no.: sc-374362, Santa Cruz Biotechnology) and anti-PSD95 (catalog no.: ab9708, Merck Millipore) overnight at 4 °C with shaking. The membranes were then washed three times for 10 minutes with TBST and incubated for 2 h at room temperature with horseradish peroxidase (HRP)-linked secondary antibodies (GE Healthcare). Finally, the membranes were washed three times for 10 minutes with TBST and enhanced chemiluminescence-based system were used for protein detection.

## Electronic supplementary material

Supplementary Files
