# Supplementary material for: Identification and characterization of the BRI2 interactome in the brain
Source: Sci Rep. 2018 Feb 23;8:3548. doi: 10.1038/s41598-018-21453-3 (PMC5824958; doi:10.1038/s41598-018-21453-3)
Supplement: Supplementary file 1 — Supplementary Files [file 41598_2018_21453_MOESM1_ESM.pdf]

Identification and characterization of the BRI2 interactome in the brain

Filipa Martins, Ana M. Marafona, Cátia D. Pereira, Thorsten Müller, Christina Loosse, Katharina Kolbe, Odete A.B. da Cruz e Silva and Sandra Rebelo

SUPLLEMENTARY FILES

SUPLLEMENTARY FIGURES

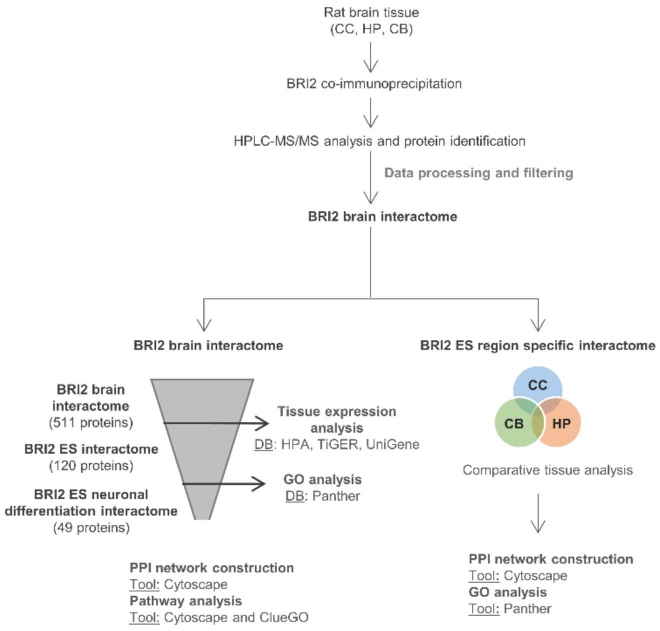

Supplementary Figure S1 – Workflow for the identification of novel brain BRI2 interacting proteins using rat brain tissue lysates. Two different approaches were used, the first one is represented on left and the second one on the right. DB, database; CC, cerebral cortex; HP, hippocampus; CB, cerebellum; MS, mass-spectrometry.

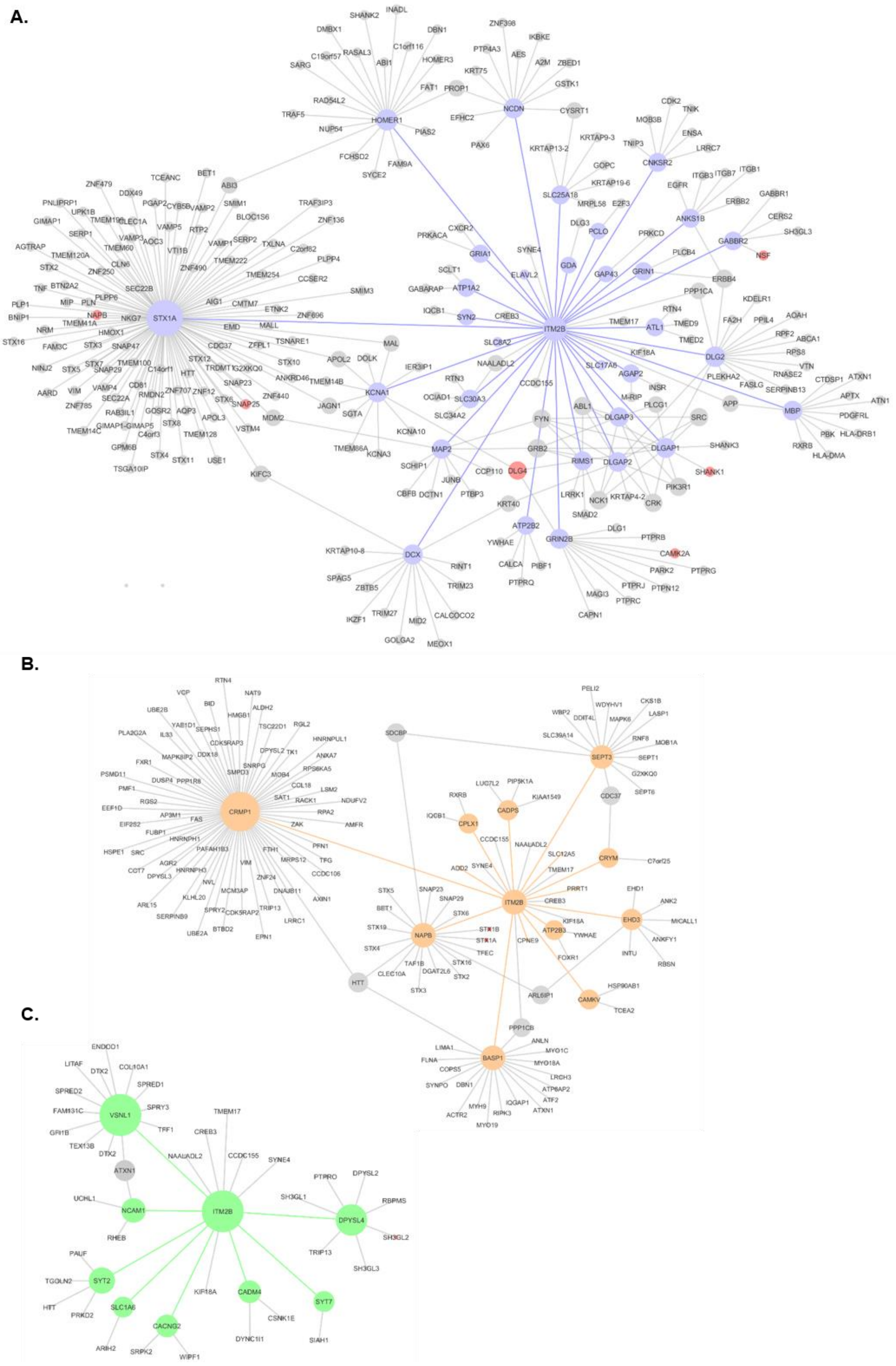

Supplementary Figure S2 – BRI2 ES-based region specific sub-networks. A- BRI2 ES-based sub-network for the cerebral cortex. Node colors represent the source of the protein: blue nodes correspond to proteins identified in this study in the cerebral cortex, grey nodes to proteins added by network augmentation, and red nodes correspond to protein added by network augmentation that are also identified in this study as BRI2 ES interactors. Node size according to the degree in the network. Edge color represent the source of interaction: blue edges correspond to the cortex specific BRI2 interactions enriched or specific for brain identified in our study, whereas the grey edges correspond to

interactions added by network augmentation. **B- BRI2 ES-based sub-network for the hippocampus.** Node colors represent the source of the protein: orange nodes correspond to proteins identified in this study in the cerebral cortex, grey nodes to proteins added by network augmentation, and red nodes correspond to protein added by network augmentation that are also identified in this study as BRI2 ES interactors. Node size according to the degree in the network. Edge color represent the source of interaction: orange edges correspond to the cortex specific BRI2 interactions enriched or specific for brain identified in our study, whereas the grey edges correspond to interactions added by network augmentation. **C- BRI2 ES-based sub-network for the cerebellum.** Node colors represent the source of the protein: green nodes correspond to proteins identified in this study in the cerebral cortex, grey nodes to proteins added by network augmentation, and red nodes correspond to protein added by network augmentation that are also identified in this study as BRI2 ES interactors. Node size according to the degree in the network. Edge color represent the source of interaction: green edges correspond to the cortex specific BRI2 interactions enriched or specific for brain identified in our study, whereas the grey edges correspond to interactions added by network augmentation.

Figure 5A (BRI2 co-immunoprecipitation)

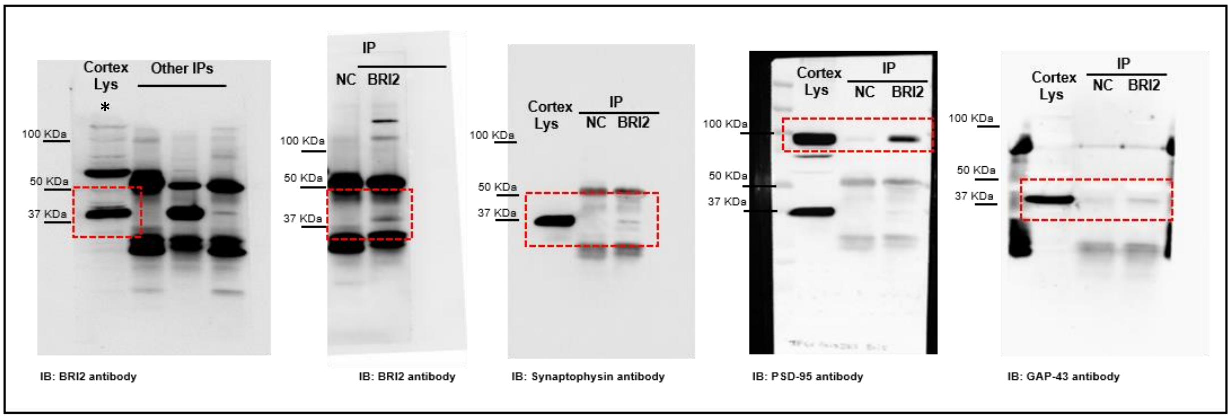

Figure 5B (PSD-95 co-immunoprecipitation)

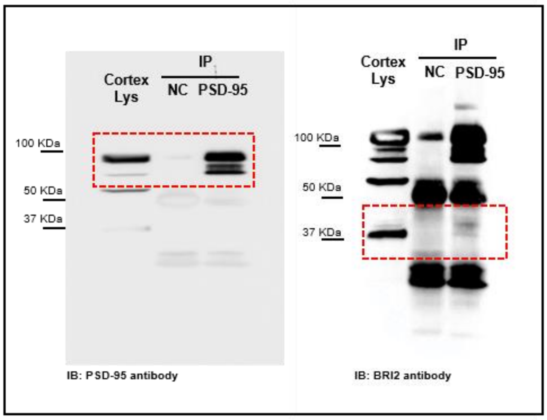

Figure 5C (Synaptophysin co-immunoprecipitation)

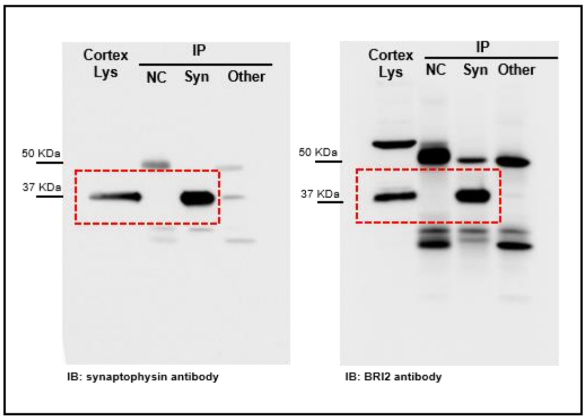

Figure 5D (GAP-43 co-immunoprecipitation)

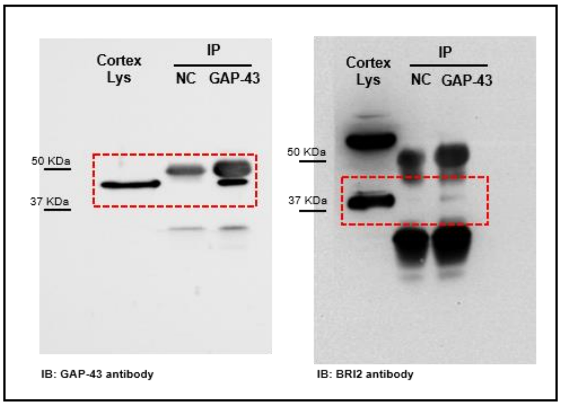

Supplementary Figure S3 – Original blots referring to main Figure 5. All immunoblots were named according to the main Figure 5. Red dotted-line boxes highlight the regions of the immunoblots selected for presentation in the main Figure 5. Lys, lysate; IP, immunoprecipitation; NC, negative control. * this cortex lysate was loaded on the same SDS-PAGE as the BRI2 immunoprecipitates, only after the transference the membranes were cut and both detected with BRI2 antibody.

SUPLLEMENTARY TABLES

Table S1 – Candidate BRI2 interacting proteins identified by Nano-HPLC-MS 7MS. Uniprot accession numbers, gene and protein names, are listed, as well as the rat brain tissues where the proteins were identified. CC, cerebral cortex; HP, hippocampus; CB, cerebellum.

Uniprot accession	Gene name	Protein name	Brain regions
P62260	Ywhae	14-3-3 protein epsilon	HP
P61983	Ywhag	14-3-3 protein gamma	CC, HP
P68255	Ywhaq	14-3-3 protein theta	CC
P63102	Ywhaz	14-3-3 protein zeta/delta	CB
P13233	Cnp	2',3'-cyclic-nucleotide 3'-phosphodiesterase	CC, HP, CB
Q4FZT9	Psmd2	26S proteasome non-ATPase regulatory subunit 2	CC, CB
P11960	Bckdha	2-oxoisovalerate dehydrogenase subunit alpha, mitochondrial	CC
O70351	Hsd17b10	3-hydroxyacyl-CoA dehydrogenase type-2	HP
P63326	Rps10	40S ribosomal protein S10	CB
P62282	Rps11	40S ribosomal protein S11	CC
P62278	Rps13	40S ribosomal protein S13	CC, CB
P62250	Rps16	40S ribosomal protein S16	CC, CB
P17074	Rps19	40S ribosomal protein S19	CC, CB
P60868	Rps20	40S ribosomal protein S20	CB
P62853	Rps25	40S ribosomal protein S25	CB
P62856	Rps26	40S ribosomal protein S26	CB
P62909	Rps3	40S ribosomal protein S3	CC, HP, CB
P49242	Rps3a	40S ribosomal protein S3a	CB
P62703	Rps4x	40S ribosomal protein S4, X isoform	CC, CB
P62083	Rps7	40S ribosomal protein S7	CB
P38983	Rpsa	40S ribosomal protein SA	CC, CB
Q794F9	Slc3a2	4F2 cell-surface antigen heavy chain	HP
P63039	Hspd1	60 kDa heat shock protein, mitochondrial	CC, HP
P19945	Rplp0	60S acidic ribosomal protein P0	CB
P62914	Rpl11	60S ribosomal protein L11	CB
P23358	Rpl12	60S ribosomal protein L12	CC, HP, CB
P41123	Rpl13	60S ribosomal protein L13	CB
P47198	Rpl22	60S ribosomal protein L22	CC
P61354	Rpl27	60S ribosomal protein L27	CC, CB
P50878	Rpl4	60S ribosomal protein L4	CB
P21533	Rpl6	60S ribosomal protein L6	CB
P05426	Rpl7	60S ribosomal protein L7	CC, CB
P06761	Hspa5	78 kDa glucose-regulated protein	CC, HP, CB
P49911	Anp32a	Acidic leucine-rich nuclear phosphoprotein 32 family member A	CC, CB
Q9ER34	Aco2	Aconitate hydratase, mitochondrial	HP
P68035	Actc1	Actin, alpha cardiac muscle 1	CC
P60711	Actb	Actin, cytoplasmic 1	CC, HP, CB
P63269	Actg2	Actin, gamma-enteric smooth muscle	CC, HP, CB
Q6KC51	Ablim2	Actin-binding LIM protein 2	CC, CB

Q4V7C7	Actr3	Actin-related protein 3	CB
P69682	Necap1	Adaptin ear-binding coat-associated protein 1	CC, HP
B5DFN2	Ahcyl1	Adenosylhomocysteinase 2	CB
P39069	Ak1	Adenylate kinase isoenzyme 1	HP
P52481	Cap2	Adenylyl cyclase-associated protein 2	CC, HP
Q05962	Slc25a4	ADP/ATP translocase 1	CC, HP
Q09073	Slc25a5	ADP/ATP translocase 2	CC, HP
P61751	Arf4	ADP-ribosylation factor 4	CC, HP
Q62848	Arfgap1	ADP-ribosylation factor GTPase-activating protein 1	CB
P07897	Acan	Aggrecan core protein	HP
P06238	A2m	Alpha-2-macroglobulin	CC, HP, CB
Q9Z1P2	Actn1	Alpha-actinin-1	CC
Q63028	Add1	Alpha-adducin	HP, CB
P85515	Actr1a	Alpha-centractin	HP
P04764	Eno1	Alpha-enolase	CC, HP
P23565	Ina	Alpha-internexin	CC, HP, CB
P54921	Napa	Alpha-soluble NSF attachment protein	HP, CB
P21396	Maoa	Amine oxidase [flavin-containing] A	HP
O08838	Amph	Amphiphysin	CC, HP, CB
P0C6S7	Anks1b	Ankyrin repeat and sterile alpha motif domain-containing protein 1B	CC
O70511	Ank3	Ankyrin-3	CC, HP, CB
P52303	Ap1b1	AP-1 complex subunit beta-1	CC, HP
Q32Q06	Ap1m1	AP-1 complex subunit mu-1	CC
P18484	Ap2a2	AP-2 complex subunit alpha-2	CC, HP, CB
P62944	Ap2b1	AP-2 complex subunit beta	CC, HP, CB
P84092	Ap2m1	AP-2 complex subunit mu	CC, HP
P62744	Ap2s1	AP-2 complex subunit sigma	CC, HP, CB
P0C1X8	Aak1	AP2-associated protein kinase 1	CC, HP
Q8CGU4	Agap2	Arf-GAP with GTPase, ANK repeat and PH domain-containing protein 2	CC
P00507	Got2	Aspartate aminotransferase, mitochondrial	CB
P15178	Dars	Aspartate--tRNA ligase, cytoplasmic	CB
Q6PST4	Atl1	Atlastin-1	CC
P19511	Atp5f1	ATP synthase F	CC, HP
P15999	Atp5a1	ATP synthase subunit alpha, mitochondrial	CC, HP
P10719	Atp5b	ATP synthase subunit beta, mitochondrial	HP
P31399	Atp5h	ATP synthase subunit d, mitochondrial	HP
P35434	Atp5d	ATP synthase subunit delta, mitochondrial	HP
P29419	Atp5i	ATP synthase subunit e, mitochondrial	CC
D3ZAF6	Atp5j2	ATP synthase subunit f, mitochondrial	HP, CB
Q6PDU7	Atp5l	ATP synthase subunit g, mitochondrial	HP, CB
P35435	Atp5c1	ATP synthase subunit gamma, mitochondrial	CC, HP, CB
Q06647	Atp5o	ATP synthase subunit O, mitochondrial	CC, HP, CB
P21571	Atp5j	ATP synthase-coupling factor 6, mitochondrial	HP
Q7TNJ2	Abca7	ATP-binding cassette sub-family A member 7	HP
P47858	Pfkm	ATP-dependent 6-phosphofructokinase, muscle type	HP
P47860	Pfkp	ATP-dependent 6-phosphofructokinase, platelet type	HP

Q9WTP0	Epb41l1	Band 4.1-like protein 1	CC, HP, CB
Q9ESS6	Bcam	Basal cell adhesion molecule	CC, HP, CB
Q05764	Add2	Beta-adducin	HP
P29066	Arrb1	Beta-arrestin-1	CC
P85969	Napb	Beta-soluble NSF attachment protein	HP
O35567	Atic	Bifunctional purine biosynthesis protein PURH Phosphoribosylaminoimidazolecarboxamide formyltransferase	CB
Q05175	Basp1	Brain acid soluble protein 1	HP
Q6GMN2	Baiap2	Brain-specific angiogenesis inhibitor 1-associated protein 2	CC
P55068	Bcan	Brevican core protein	HP, CB
P11275	Camk2a	Calcium/calmodulin-dependent protein kinase type II subunit alpha	CC, HP
P08413	Camk2b	Calcium/calmodulin-dependent protein kinase type II subunit beta	CC, HP, CB
P15791	Camk2d	Calcium/calmodulin-dependent protein kinase type II subunit delta	CC, CB
P11730	Camk2g	Calcium/calmodulin-dependent protein kinase type II subunit gamma	CC, CB
Q66HR5	Calcoco1	Calcium-binding and coiled-coil domain-containing protein 1	CC, HP
Q62717	Cadps	Calcium-dependent secretion activator 1	HP
P62161	Calm1	Calmodulin	HP, CB
Q07009	Capn2	Calpain-2 catalytic subunit	CB
Q63092	Camkv	CaM kinase-like vesicle-associated protein	HP
P27791	Prkaca	cAMP-dependent protein kinase catalytic subunit alpha	CC
P48284	Ca4	Carbonic anhydrase 4	CB
P19139	Csnk2a1	Casein kinase II subunit alpha	CC, HP, CB
P67874	Csnk2b	Casein kinase II subunit beta	CC
Q9WU82	Ctnnb1	Catenin beta-1	CC, CB
B5D5N9	Slc7a2	Cationic amino acid transporter 2	CC, HP
Q5M7A7	Cnrip1	CB1 cannabinoid receptor-interacting protein 1	CB
P40241	Cd9	CD9 antigen	CC, HP, CB
B0K020	Cisd1	CDGSH iron-sulfur domain-containing protein 1	CC
Q1WIM1	Cadm4	Cell adhesion molecule 4	CB
Q5FVI4	Cend1	Cell cycle exit and neuronal differentiation protein 1	CC, HP
B2RYL1	Chtf8	Chromosome transmission fidelity protein 8 homolog isoform 2	CC
Q8VHF5	Cs	Citrate synthase, mitochondrial	CC
Q05140	Snap91	Clathrin coat assembly protein AP180	CC, HP
P11442	Cltc	Clathrin heavy chain 1	CC, HP
P08081	Clta	Clathrin light chain A	CC, HP
P08082	Cltb	Clathrin light chain B	CC, HP
Q99P82	Cldn11	Claudin-11	CC
Q99JD4	Clasp2	CLIP-associating protein 2	CB
P45592	Cfl1	Cofilin-1	CC, HP, CB
P13941	Col3a1	Collagen alpha-1	CB
P31720	C1qa	Complement C1q subcomponent subunit A	HP
P31721	C1qb	Complement C1q subcomponent subunit B	CC, HP, CB
P31722	C1qc	Complement C1q subcomponent subunit C	CC
P01026	C3	Complement C3 [Cleaved into: Complement C3 beta chain; C3-beta-c	CC, HP
O35796	C1qbp	Complement component 1 Q subcomponent-binding protein, mitochondrial	CB
P63041	Cplx1	Complexin-1	HP
Q9Z1T4	Cnksr2	Connector enhancer of kinase suppressor of ras 2	CC

Q63198	Cntn1	Contactin-1	CC, HP
P97846	Cntnap1	Contactin-associated protein 1	CC, CB
Q5BJS7	Cpne9	Copine-9	HP
Q02874	H2afy	Core histone macro-H2A.1	CB
P48199	Crp	C-reactive protein	CB
P00564	Ckm	Creatine kinase M-type	CB
P25809	Ckmt1	Creatine kinase U-type, mitochondrial	CC, HP, CB
P97536	Cand1	Cullin-associated NEDD8-dissociated protein 1	CB
O08565	Cxcr4	C-X-C chemokine receptor type 4	HP
Q03114	Cdk5	Cyclin-dependent-like kinase 5	CC
P97874	Gak	Cyclin-G-associated kinase	CC, HP
P32551	Uqcrc2	Cytochrome b-c1 complex subunit 2, mitochondrial	CC, HP, CB
Q7TQ16	Uqcrq	Cytochrome b-c1 complex subunit 8	CC, HP, CB
P20788	Uqcrfs1	Cytochrome b-c1 complex subunit Rieske, mitochondrial	HP, CB
P00406	Mtco2	Cytochrome c oxidase subunit 2	HP
P10888	Cox4i1	Cytochrome c oxidase subunit 4 isoform 1, mitochondrial	CC, HP
P11240	Cox5a	Cytochrome c oxidase subunit 5A, mitochondrial	CB
P12075	Cox5b	Cytochrome c oxidase subunit 5B, mitochondrial	CC, HP
P11951	Cox6c2	Cytochrome c oxidase subunit 6C-2	HP
P35171	Cox7a2	Cytochrome c oxidase subunit 7A2, mitochondrial	HP
P38650	Dync1h1	Cytoplasmic dynein 1 heavy chain 1	CC, HP
Q6Q0N1	Cndp2	Cytosolic non-specific dipeptidase	CB
O08651	Phgdh	D-3-phosphoglycerate dehydrogenase	CC, CB
P29147	Bdh1	D-beta-hydroxybutyrate dehydrogenase, mitochondrial	CC, HP
P08461	Dlat	Dihydrolipoyllysine-residue acetyltransferase component of pyruvate dehydrogenase complex, mitochondrial	CC
Q01205	Dlst	Dihydrolipoyllysine-residue succinyltransferase component of 2-oxoglutarate dehydrogenase complex, mitochondrial	HP, CB
Q62950	Crmp1	Dihydropyrimidinase-related protein 1	HP
P47942	Dpysl2	Dihydropyrimidinase-related protein 2	CC, HP
Q62951	Dpysl4	Dihydropyrimidinase-related protein 4	CB
Q63342	Dmgdh	Dimethylglycine dehydrogenase, mitochondrial	CC
Q62696	Dlg1	Disks large homolog 1	CC, CB
Q63622	Dlg2	Disks large homolog 2	CC
P31016	Dlg4	Disks large homolog 4	CC, HP, CB
P97836	Dlgap1	Disks large-associated protein 1	CC
P97837	Dlgap2	Disks large-associated protein 2	CC
P97838	Dlgap3	Disks large-associated protein 3	CC
Q07266	Dbn1	Drebrin	CC, HP
Q01986	Map2k1	Dual specificity mitogen-activated protein kinase kinase 1	HP, CB
Q6AYH5	Dctn2	Dynactin subunit 2	CC, HP
P21575	Dnm1	Dynamin-1	CC, HP
O35303	Dnm1l	Dynamin-1-like protein	CC, HP
Q2TA68	Opa1	Dynamin-like 120 kDa protein, mitochondrial	CC
B0BND0	Enpp6	Ectonucleotide pyrophosphatase/phosphodiesterase family member 6	CB
Q8R491	Ehd3	EH domain-containing protein 3	HP
Q8CH84	Elavl2	ELAV-like protein 2	CC
Q9JI66	Slc4a4	Electrogenic sodium bicarbonate cotransporter 1	HP, CB

P62630	Eef1a1	Elongation factor 1-alpha 1	CC, HP
Q68FR6	Eef1g	Elongation factor 1-gamma	CB
P05197	Eef2	Elongation factor 2	CC, HP, CB
P85834	Tufm	Elongation factor Tu, mitochondrial	CC, HP, CB
O35179	Sh3gl2	Endophilin-A1	CC, CB
Q5PPJ9	Sh3glb2	Endophilin-B2	CC, HP, CB
Q66HD0	Hsp90b1	Endoplasmin	CC, HP, CB
O88339	Epn1	Epsin-1	CC
B5DEH2	Erlin2	Erlin-2	CB
Q5RKI1	Eif4a2	Eukaryotic initiation factor 4A-II	HP, CB
P24942	Slc1a3	Excitatory amino acid transporter 1	CC, HP, CB
P31596	Slc1a2	Excitatory amino acid transporter 2	CC, HP, CB
O35921	Slc1a6	Excitatory amino acid transporter 4	CB
B2GUZ5	Capza1	F-actin-capping protein subunit alpha-1	CB
Q3T1K5	Capza2	F-actin-capping protein subunit alpha-2	CC
Q5XI32	Capzb	F-actin-capping protein subunit beta	CC, CB
P12785	Fasn	Fatty acid synthase	HP
Q5XI81	Fxr1	Fragile X mental retardation syndrome-related protein 1	CB
P05065	Aldoa	Fructose-bisphosphate aldolase A	CC, HP
O88871	Gabbr2	Gamma-aminobutyric acid type B receptor subunit 2	CC
P08050	Gja1	Gap junction alpha-1 protein	CC, HP, CB
Q68FP1	Gsn	Gelsolin	CC, CB
Q63228	Gmfb	Glia maturation factor beta	CC
P47819	Gfap	Glial fibrillary acidic protein	CC, HP, CB
P19490	Gria1	Glutamate receptor 1	CC
P19491	Gria2	Glutamate receptor 2	CC, HP, CB
Q63226	Grid2	Glutamate receptor ionotropic, delta-2	CB
P35439	Grin1	Glutamate receptor ionotropic, NMDA 1	CC
Q00960	Grin2b	Glutamate receptor ionotropic, NMDA 2B	CC
P28492	Gls2	Glutaminase liver isoform, mitochondrial	HP
P09606	Glul	Glutamine synthetase	CC, HP, CB
P04797	Gapdh	Glyceraldehyde-3-phosphate dehydrogenase	CC
P35571	Gpd2	Glycerol-3-phosphate dehydrogenase, mitochondrial	HP
P53534	Pygb	Glycogen phosphorylase, brain form	CC, HP
P08644	Kras	GTPase KRas	CC
Q63942	Rab3d	GTP-binding protein Rab-3D	HP
Q9WTT6	Gda	Guanine deaminase	CC
P43425	Gng7	Guanine nucleotide-binding protein G(I)/G(S)/G(O) subunit gamma-7	CC
P63095	Gnas	Guanine nucleotide-binding protein G(s) subunit alpha isoforms short	CC
P52287	Gnb3	Guanine nucleotide-binding protein G(I)/G(S)/G(T) subunit beta-3	CC
P82471	Gnaq	Guanine nucleotide-binding protein G(q) subunit alpha	CB
P54311	Gnb1	Guanine nucleotide-binding protein G(I)/G(S)/G(T) subunit beta-1	CC, HP
P10824	Gnai1	Guanine nucleotide-binding protein G(i) subunit alpha-1	CC, HP
P19627	Gnaz	Guanine nucleotide-binding protein G(z) subunit alpha	CC, HP
P59215	Gnao1	Guanine nucleotide-binding protein G(o) subunit alpha	CC, HP, CB
P04897	Gnai2	Guanine nucleotide-binding protein G(i) subunit alpha-2	CC, HP, CB

O35353	Gnb4	Guanine nucleotide-binding protein subunit beta-4	HP, CB
P55063	Hspa1l	Heat shock 70 kDa protein 1-like	CC
O88600	Hspa4	Heat shock 70 kDa protein 4	CB
P63018	Hspa8	Heat shock cognate 71 kDa protein	CC
P82995	Hsp90aa1	Heat shock protein HSP 90-alpha	CC, HP, CB
P34058	Hsp90ab1	Heat shock protein HSP 90-beta	CC, HP, CB
P11517	ND	Hemoglobin subunit beta-2	CB
Q6URK4	Hnrnpa3	Heterogeneous nuclear ribonucleoprotein A3	CC, CB
G3V9R8	Hnrnpc	Heterogeneous nuclear ribonucleoprotein C	CC, CB
Q9JJ54	Hnrnpd	Heterogeneous nuclear ribonucleoprotein D0	CC, CB
Q3SWU3	Hnrnpdl	Heterogeneous nuclear ribonucleoprotein D-like	CC, HP, CB
Q794E4	Hnrnpf	Heterogeneous nuclear ribonucleoprotein F	CC, CB
Q8VHV7	Hnrnph1	Heterogeneous nuclear ribonucleoprotein H	CC
Q6AY09	Hnrnph2	Heterogeneous nuclear ribonucleoprotein H2	CC, HP, CB
P61980	Hnrnpk	Heterogeneous nuclear ribonucleoprotein K	CC, HP, CB
Q62826	Hnrnpm	Heterogeneous nuclear ribonucleoprotein M	CB
Q7TP47	Syncrip	Heterogeneous nuclear ribonucleoprotein Q	CB
A7VJC2	Hnrnpa2b1	Heterogeneous nuclear ribonucleoproteins A2/B1	CC, CB
P05708	Hk1	Hexokinase-1	CC, HP
P02262	ND	Histone H2A type 1	CC, CB
P0C0S7	H2afz	Histone H2A.Z	CC, HP
Q00715	ND	Histone H2B type 1	CC, HP, CB
P62804	Hist1h4b	Histone H4	CC, HP, CB
Q9Z214	Homer1	Homer protein homolog 1	CC
Q9Z2X5	Homer3	Homer protein homolog 3	CB
P03994	Hapln1	Hyaluronan and proteoglycan link protein 1	CC, HP, CB
Q9ESM2	Hapln2	Hyaluronan and proteoglycan link protein 2	CC, CB
Q9WVK7	Hadh	Hydroxyacyl-coenzyme A dehydrogenase, mitochondrial	CC, HP
Q63617	Hyou1	Hypoxia up-regulated protein 1	CC, HP, CB
P20761	Igh-1a	Ig gamma-2B chain C region	CC, HP
P29994	Itpr1	Inositol 1,4,5-trisphosphate receptor type 1	CB
Q63269	Itpr3	Inositol 1,4,5-trisphosphate receptor type 3	CB
D3ZGS3	Ocrl	Inositol polyphosphate 5-phosphatase OCRL-1	CC, HP
Q63416	Itih3	Inter-alpha-trypsin inhibitor heavy chain H3	CC, HP
Q7TP98	Ilf2	Interleukin enhancer-binding factor 2	CB
Q9WVE9	Itsn1	Intersectin-1	CC, HP
Q99NA5	Idh3a	Isocitrate dehydrogenase [NAD] subunit alpha, mitochondrial	CC, HP
Q68FX0	Idh3B	Isocitrate dehydrogenase [NAD] subunit beta, mitochondrial	CC, HP, CB
P41565	Idh3g	Isocitrate dehydrogenase [NAD] subunit gamma 1, mitochondrial	CC
P56574	Idh2	Isocitrate dehydrogenase [NADP], mitochondrial	CB
Q9QYU4	Crym	Ketimine reductase mu-crystallin	HP
Q6QLM7	Kif5a	Kinesin heavy chain isoform 5A	CC, HP, CB
P56536	Kif5c	Kinesin heavy chain isoform 5C	CC, CB
P37285	Klc1	Kinesin light chain 1	CC, HP, CB
Q2PQA9	Kif5b	Kinesin-1 heavy chain	CC, HP, CB
Q9WV63	Kif2a	Kinesin-like protein KIF2A	CB

Q5XI51	Kif2b	Kinesin-like protein KIF2B	CC
P70615	Lmnb1	Lamin-B1	CB
Q5XIN6	Letm1	LETM1 and EF-hand domain-containing protein 1, mitochondrial	CB
P97829	Cd47	Leukocyte surface antigen CD47	CC, HP
Q62813	Lsamp	Limbic system-associated membrane protein	CC, CB
P04642	Ldha	L-lactate dehydrogenase A chain	HP
P04636	Mdh2	Malate dehydrogenase, mitochondrial	HP
O08873	Madd	MAP kinase-activating death domain protein	HP
P43244	Matr3	Matrin-3	CC, HP
Q4L1J4	Magi1	Membrane-associated guanylate kinase, WW and PDZ domain-containing protein 1	HP
Q00566	Mecp2	Methyl-CpG-binding protein 2	CB
D4A7N1	Chchd6	MICOS complex subunit Mic25	CC, CB
Q3KR86	Immt	MICOS complex subunit Mic60	CC, HP, CB
D3ZHV2	Macf1	Microtubule-actin cross-linking factor 1	CC, CB
P34926	Map1a	Microtubule-associated protein 1A	CC, HP
P15205	Map1b	Microtubule-associated protein 1B	CC, HP, CB
P15146	Map2	Microtubule-associated protein 2	CC
Q5M7W5	Map4	Microtubule-associated protein 4	CC, HP
Q63560	Map6	Microtubule-associated protein 6	CC, HP, CB
F1LP90	Mink1	Misshapen-like kinase 1	CC, HP
P97700	Slc25a11	Mitochondrial 2-oxoglutarate/malate carrier protein	CC, HP, CB
Q505J6	Slc25a18	Mitochondrial glutamate carrier 2	CC
O08839	Bin1	Myc box-dependent-interacting protein 1	CC, HP
P02688	Mbp	Myelin basic protein	CC
P60203	Plp1	Myelin proteolipid protein	CC, CB
P07722	Mag	Myelin-associated glycoprotein	CC, CB
Q63345	Mog	Myelin-oligodendrocyte glycoprotein	CC, CB
Q6VBQ5	Myadm	Myeloid-associated differentiation marker	CC
Q9QZ76	Mb	Myoglobin	HP
P02600	Myl1	Myosin light chain 1/3, skeletal muscle isoform	HP
P16409	Myl3	Myosin light chain 3	HP, CB
Q64119	Myl6	Myosin light polypeptide 6	CC, HP, CB
P13832	Rlc-a	Myosin regulatory light chain RLC-A	CC, CB
Q64122	Myl9	Myosin regulatory light polypeptide 9	CC
Q9JLT0	Myh10	Myosin-10	CC, CB
Q62812	Myh9	Myosin-9	CC, CB
Q5RJQ4	Sirt2	NAD-dependent protein deacetylase sirtuin-2	CC, CB
Q561S0	Ndufa10	NADH dehydrogenase [ubiquinone] 1 alpha subcomplex subunit 10, mitochondrial	HP, CB
Q5BK63	Ndufa9	NADH dehydrogenase [ubiquinone] 1 alpha subcomplex subunit 9, mitochondrial	HP
P19234	Ndufv2	NADH dehydrogenase [ubiquinone] flavoprotein 2, mitochondrial	HP, CB
Q641Y2	Ndufs2	NADH dehydrogenase [ubiquinone] iron-sulfur protein 2, mitochondrial	HP
P52504	Ndufs6	NADH dehydrogenase [ubiquinone] iron-sulfur protein 6, mitochondrial	HP
Q66HF1	Ndufs1	NADH-ubiquinone oxidoreductase 75 kDa subunit, mitochondrial	CC, HP, CB
O35867	Ppp1r9a	Neurabin-1	CC
P13596	Ncam1	Neural cell adhesion molecule 1	CB

P55067	Ncan	Neurocan core protein	CC, HP, CB
O35095	Ncdn	Neurochondrin	CC
P97685	Nfasc	Neurofascin	CC, HP, CB
P16884	Nefh	Neurofilament heavy polypeptide	CC, HP, CB
P19527	Nefl	Neurofilament light polypeptide	CC, HP, CB
P12839	Nefm	Neurofilament medium polypeptide	CC, CB
P07936	Gap43	Neuromodulin	CC
Q9ESI7	Dcx	Neuronal migration protein doublecortin	CC
Q9WU34	Sept3	Neuronal-specific septin-3	HP
P97546	Nptn	Neuroplastin	CC
Q62718	Ntm	Neurotrimin	CC, CB
P08460	Nid1	Nidogen-1	CB
Q9EPI6	Nsmf	NMDA receptor synaptonuclear signaling and neuronal migration factor	CC
A1L1I3	Numbl	Numb-like protein	CC
P04218	Cd200	OX-2 membrane glycoprotein	CC
P10111	Ppia	Peptidyl-prolyl cis-trans isomerase A	CB
Q9QVC8	Fkbp4	Peptidyl-prolyl cis-trans isomerase FKBP4	CB
Q63716	Prdx1	Peroxiredoxin-1	CC, HP
P35704	Prdx2	Peroxiredoxin-2	CC
O35244	Prdx6	Peroxiredoxin-6	CC, HP
Q642G4	Pex14	Peroxisomal membrane protein PEX14	CC
P16036	Slc25a3	Phosphate carrier protein, mitochondrial	CC, HP, CB
O08662	Pi4ka	Phosphatidylinositol 4-kinase alpha	CC
P16617	Pgk1	Phosphoglycerate kinase 1	HP
P16290	Pgam2	Phosphoglycerate mutase 2	CB
P11505	Atp2b1	Plasma membrane calcium-transporting ATPase 1	CC
P11506	Atp2b2	Plasma membrane calcium-transporting ATPase 2	CC
Q64568	Atp2b3	Plasma membrane calcium-transporting ATPase 3	HP
Q64542	Atp2b4	Plasma membrane calcium-transporting ATPase 4	CC
P30427	Plec	Plectin	CC, CB
Q9EPH8	Pabpc1	Polyadenylate-binding protein 1	CB
P0CG51	Ubb	Polyubiquitin-B [Cleaved into: Ubiquitin]	CC
P10499	Kcna1	Potassium voltage-gated channel subfamily A member 1	CC
P48679	Lmna	Prelamin-A/C [Cleaved into: Lamin-A/C]	CB
Q9JMJ4	Prpf19	Pre-mRNA-processing factor 19	HP
P62963	Pfn1	Profilin-1	CC
P67779	Phb	Prohibitin	CC, HP, CB
Q5XIH7	Phb2	Prohibitin-2	CC, HP, CB
P04961	Pcna	Proliferating cell nuclear antigen	CB
Q6AYD3	Pa2g4	Proliferation-associated protein 2G4	CC
Q6MG82	Prrt1	Proline-rich transmembrane protein 1	HP
O88778	Bsn	Protein bassoon	CC, CB
O88767	Park7	Protein deglycase DJ-1	CC
P04785	P4hb	Protein disulfide-isomerase	CB
P63319	Prkcg	Protein kinase C gamma type	CC, HP, CB
Q8VBU2	Ndrg2	Protein NDRG2	HP

Q9JKS6	Pclo	Protein piccolo	CC
Q6MG48	Prrc2a	Protein PRRC2A	CC
P23606	Tgm1	Protein-glutamine gamma-glutamyltransferase K	CB
P52873	Pc	Pyruvate carboxylase, mitochondrial	HP, CB
P26284	Pdha1	Pyruvate dehydrogenase E1 component subunit alpha, somatic form, mitochondrial	CC, CB
P49432	Pdhb	Pyruvate dehydrogenase E1 component subunit beta, mitochondrial	CC, HP, CB
P11980	Pkm	Pyruvate kinase PKM	CC, HP
P50399	Gdi2	Rab GDP dissociation inhibitor beta	HP, CB
P47709	Rph3a	Rabphilin-3A	CC, HP
F1M386	Rapgef2	Rap guanine nucleotide exchange factor 2	CC
Q9QUH6	Syngap1	Ras/Rap GTPase-activating protein SynGAP	CC
Q6RUV5	Rac1	Ras-related C3 botulinum toxin substrate 1	CC, CB
P35281	Rab10	Ras-related protein Rab-10	CC, HP
P61107	Rab14	Ras-related protein Rab-14	CC, CB
Q6NYB7	Rab1A	Ras-related protein Rab-1A	CC, CB
P10536	Rab1b	Ras-related protein Rab-1B	CB
P05712	Rab2a	Ras-related protein Rab-2A	CC
P63012	Rab3a	Ras-related protein Rab-3A	CC, HP, CB
Q63941	Rab3b	Ras-related protein Rab-3B	CC
P62824	Rab3c	Ras-related protein Rab-3C	CC, HP
M0RC99	Rab5a	Ras-related protein Rab-5A	CB
P09527	Rab7a	Ras-related protein Rab-7a	CB
P61227	Rap2b	Ras-related protein Rap-2b	HP
P63245	Rack1	Receptor of activated protein C kinase 1	CC, CB
Q6AXX6	Fam213a	Redox-regulatory protein FAM213A	CC, HP
Q9JIR4	Rims1	Regulating synaptic membrane exocytosis protein 1	CC
Q62703	Rcn2	Reticulocalbin-2	CB
Q64548	Rtn1	Reticulon-1	CC, HP, CB
Q6RJR6	Rtn3	Reticulon-3	CC, HP, CB
Q9JK11	Rtn4	Reticulon-4	CC
D4AE41	Rbmxl1	RNA binding motif protein, X-linked-like-1	CC, CB
Q3B7K9	Rundc3b	RUN domain-containing protein 3B	CC, HP
Q64578	Atp2a1	Sarcoplasmic/endoplasmic reticulum calcium ATPase 1	CC, CB
P11507	Atp2a2	Sarcoplasmic/endoplasmic reticulum calcium ATPase 2	HP
O88453	Safb	Scaffold attachment factor B1	CB
P56603	Scamp1	Secretory carrier-associated membrane protein 1	CC, HP
Q9JKE3	Scamp5	Secretory carrier-associated membrane protein 5	CC, HP
B3GNI6	Sept11	Septin-11	CC, CB
Q9JJM9	Sept5	Septin-5	CC, HP
Q9WVC0	Sept7	Septin-7	CB
G3V6S8	Srsf6	Serine/arginine-rich splicing factor 6	CB
O08875	Dclk1	Serine/threonine-protein kinase DCLK1	CC, HP
O08679	Mark2	Serine/threonine-protein kinase MARK2	CC
Q4QQT4	Ppp2r1b	Serine/threonine-protein phosphatase 2A 65 kDa regulatory subunit A beta isoform	CC
P63329	Ppp3ca	Serine/threonine-protein phosphatase 2B catalytic subunit alpha isoform	CC
Q64620	Ppp6c	Serine/threonine-protein phosphatase 6 catalytic subunit	CC

Q562B5	Pgam5	Serine/threonine-protein phosphatase PGAM5, mitochondrial	CC
P62138	Ppp1ca	Serine/threonine-protein phosphatase PP1-alpha catalytic subunit	CC
Q6P799	Sars	Serine--tRNA ligase, cytoplasmic	CB
P12346	Tf	Serotransferrin	CC, HP, CB
P23680	Apcs	Serum amyloid P-component	CB
Q9WV48	Shank1	SH3 and multiple ankyrin repeat domains protein 1	CC, CB
Q9QX74	Shank2	SH3 and multiple ankyrin repeat domains protein 2	CC, CB
Q9JLU4	Shank3	SH3 and multiple ankyrin repeat domains protein 3	CC
P0DJJ3	Sgip1	SH3-containing GRB2-like protein 3-interacting protein 1	CC, HP
Q63965	Sfxn1	Sideroflexin-1	CC, HP
Q9JHY2	Sfxn3	Sideroflexin-3	CC, CB
P31647	Slc6a11	Sodium- and chloride-dependent GABA transporter 3	CC, CB
P48768	Slc8a2	Sodium/calcium exchanger 2	CC
P06685	Atp1a1	Sodium/potassium-transporting ATPase subunit alpha-1	CC
P06686	Atp1a2	Sodium/potassium-transporting ATPase subunit alpha-2	CC
P06687	Atp1a3	Sodium/potassium-transporting ATPase subunit alpha-3	CC, HP
P07340	Atp1b1	Sodium/potassium-transporting ATPase subunit beta-1	CC, HP, CB
P13638	Atp1b2	Sodium/potassium-transporting ATPase subunit beta-2	CC, HP, CB
Q63633	Slc12a5	Solute carrier family 12 member 5	HP
Q66HR0	Slc12a9	Solute carrier family 12 member 9	HP
P11167	Slc2a1	Solute carrier family 2, facilitated glucose transporter member 1	CC, HP, CB
O35413	Sorbs2	Sorbin and SH3 domain-containing protein 2	CC
P16086	Sptan1	Spectrin alpha chain, non-erythrocytic 1	CC, HP, CB
Q9QWN8	Sptbn2	Spectrin beta chain, non-erythrocytic 2	CC, CB
Q9QXY2	Srcin1	SRC kinase signaling inhibitor 1	CC, CB
Q4FZT0	Stoml2	Stomatin-like protein 2, mitochondrial	CC
D4AB66	Ston2	Stonin-2	CC, HP
P21913	Sdhb	Succinate dehydrogenase [ubiquinone] iron-sulfur subunit, mitochondrial	CB
P13086	Suclg1	Succinate--CoA ligase [ADP/GDP-forming] subunit alpha, mitochondrial	CB
P09951	Syn1	Synapsin-1	CC, HP
Q63537	Syn2	Synapsin-2	CC
Q02563	Sv2a	Synaptic vesicle glycoprotein 2A	CC, HP, CB
Q63564	Sv2b	Synaptic vesicle glycoprotein 2B	CC, HP
Q62876	Syngr1	Synaptogyrin-1	CC, CB
Q62910	Synj1	Synaptojanin-1	CC, HP
P07825	Syp	Synaptophysin	CC, HP
Q9Z327	Synpo	Synaptopodin	CC
P60881	Snap25	Synaptosomal-associated protein 25	HP, CB
P21707	Syt1	Synaptotagmin-1	CC, HP, CB
P97610	Syt12	Synaptotagmin-12	CC, CB
P29101	Syt2	Synaptotagmin-2	CB
Q62747	Syt7	Synaptotagmin-7	CB
P32851	Stx1a	Syntaxin-1A	CC
P61265	Stx1b	Syntaxin-1B	CC, HP, CB
P61765	Stxbp1	Syntaxin-binding protein 1	CC, HP
Q5XIM9	Cct2	T-complex protein 1 subunit beta	HP, CB

Q7TPB1	Cct4	T-complex protein 1 subunit delta	HP, CB
Q68FQ0	Cct5	T-complex protein 1 subunit epsilon	CC, HP, CB
Q6P502	Cct3	T-complex protein 1 subunit gamma	HP, CB
Q05546	Tnr	Tenascin-R	CC, HP, CB
Q920J4	Txnl1	Thioredoxin-like protein 1	HP
P01830	Thy1	Thy-1 membrane glycoprotein	HP, CB
Q5U1Z2	Trappc3	Trafficking protein particle complex subunit 3	HP
P86252	Pura	Transcriptional activator protein Pur-alpha	CC, HP, CB
Q68A21	Purb	Transcriptional activator protein Pur-beta	CC, HP, CB
Q5M7W4	Tmc5	Transmembrane channel-like protein 5	HP
Q64428	Hadha	Trifunctional enzyme subunit alpha, mitochondrial	CC
Q60587	Hadhb	Trifunctional enzyme subunit beta, mitochondrial	HP
P48500	Tpi1	Triosephosphate isomerase	CC
Q6AYT3	Rtcb	tRNA-splicing ligase RtcB homolog	CB
P70566	Tmod2	Tropomodulin-2	CC, CB
Q63610	Tpm3	Tropomyosin alpha-3 chain	CC
P09495	Tpm4	Tropomyosin alpha-4 chain	HP
Q6P7B0	Wars	Tryptophan--tRNA ligase, cytoplasmic	CC, HP
P68370	Tuba1a	Tubulin alpha-1A chain	CC, HP, CB
Q6AYZ1	Tuba1c	Tubulin alpha-1C chain	HP
Q68FR8	Tuba3a	Tubulin alpha-3 chain	CC
Q5XIF6	Tuba4a	Tubulin alpha-4A chain	CC, HP, CB
Q6AY56	Tuba8	Tubulin alpha-8 chain	CB
P85108	Tubb2a	Tubulin beta-2A chain	CC, HP, CB
Q3KRE8	Tubb2b	Tubulin beta-2B chain	CC, HP
Q4QRB4	Tubb3	Tubulin beta-3 chain	CC, HP, CB
Q6P9T8	Tubb4b	Tubulin beta-4B chain	CC, HP, CB
P69897	Tubb5	Tubulin beta-5 chain	CC, HP, CB
B2RYG6	Otub1	Ubiquitin thioesterase OTUB1	HP
Q63357	Myo1d	Unconventional myosin-Id	CC
Q9QYF3	Myo5a	Unconventional myosin-Va	CC, CB
Q9JJW3	Usmg5	Up-regulated during skeletal muscle growth protein 5	CC, HP, CB
Q793F9	Vps4a	Vacuolar protein sorting-associated protein 4A	CB
Q04462	Vars	Valine--tRNA ligase	CB
Q9ERB4	Vcan	Versican core protein	CB
Q63666	Vamp1	Vesicle-associated membrane protein 1	CB
P63045	Vamp2	Vesicle-associated membrane protein 2	CB
P63025	Vamp3	Vesicle-associated membrane protein 3	CC, CB
Q9Z270	Vapa	Vesicle-associated membrane protein-associated protein A	HP, CB
Q9QUL6	Nsf	Vesicle-fusing ATPase	CC, HP, CB
Q62634	Slc17a7	Vesicular glutamate transporter 1	CC, HP
Q9JI12	Slc17a6	Vesicular glutamate transporter 2	CC
O35458	Slc32a1	Vesicular inhibitory amino acid transporter	CC, HP
P31000	Vim	Vimentin	CC, CB
P62762	Vsnl1	Visinin-like protein 1	CB
Q9Z2L0	Vdac1	Voltage-dependent anion-selective channel protein 1	CC, HP

P81155	Vdac2	Voltage-dependent anion-selective channel protein 2	CC, HP
Q9R1Z0	Vdac3	Voltage-dependent anion-selective channel protein 3	CC, HP, CB
Q71RJ2	Cacng2	Voltage-dependent calcium channel gamma-2 subunit	CB
P54290	Cacna2d1	Voltage-dependent calcium channel subunit alpha-2/delta-1	CC
P54287	Cacnb3	Voltage-dependent L-type calcium channel subunit beta-3	HP
Q5J3M4	Vom1r94	Vomeronasal type-1 receptor 94	CC
P25286	Atp6v0a1	V-type proton ATPase 116 kDa subunit a isoform 1	CC, HP, CB
P63081	Atp6v0c	V-type proton ATPase 16 kDa proteolipid subunit	CC, HP, CB
P62815	Atp6v1b2	V-type proton ATPase subunit B, brain isoform	CC
Q6PCU2	Atp6v1e1	V-type proton ATPase subunit E 1	HP
O54715	Atp6ap1	V-type proton ATPase subunit S1	CC
Q9ERH3	Wdr7	WD repeat-containing protein 7	CC
Q6QIX3	Slc30a3	Zinc transporter 3	CC

Table S2 – Panther Protein class analysis of all candidate BRI2 interactors identified in this study. In the table are listed the Panther protein class terms and the respective IDs, as well as the Uniprot accessions of the proteins that belong to each class.

**Panther Protein Class (term ID)**	**Uniprot accessions of mapped proteins**
ATP synthase (PC00227)	P15999, Q06647, P25286, O54715, D3ZAF6, P62815, P10719, P63081
G-protein (PC00095)	B3GNI6, P62630, P61227, P21575, O35303, P08644, P10824, P61751, P54311, P59215, P82471, P05197, Q9JJM9, P19627, Q9WVC0, Q6RUV5, Q6PST4, P85834, P43425, O35353, Q9WU34, Q2TA68, P04897, P52287
actin family cytoskeletal protein (PC00085)	P0DJJ3, O35413, P63269, Q05764, P09495, Q9QYF3, P45592, B2GUZ5, Q64122, P85515, P68035, Q64119, Q3T1K5, Q63610, Q5XI32, Q9Z1P2, P16409, P52481, O08839, D3ZHV2, P70566, P30427, P16086, Q9JLT0, O08838, Q07266, P13832, Q68FP1, Q4V7C7, Q9QWN8, Q6KC51, P60711, Q62812, Q9Z327, Q63028, P09951, P02600, Q63357, Q63537
anion channel (PC00002)	P15999, P81155, Q9R1Z0, P62815, P10719, Q9Z2L0
calcium-binding protein (PC00060)	Q9QVC8, P62161, P97700, Q8R491, Q05962, P62161, Q64122, Q505J6, Q07009, Q5XIN6, Q64119, Q64620, P16409, Q62717, P63319, P62161, P62138, P13832, Q62703, Q9WVE9, P63329, Q09073, P02600, P16036
cation transporter (PC00227)	P15999, P06687, Q06647, Q9JI12, Q9JHY2, P11507, P13638, P11505, Q63965, P25286, P06685, P48768, O54715, D3ZAF6, O35921, P07340, P11506, P62815, Q62634, Q64542, P06686, Q64568, P31596, Q9JI66, P10719, P63081, P24942, Q64578, P31647
chaperone (PC00072)	P68255, Q9QVC8, P82995, Q7TPB1, P97874, Q6P502, P34058, P63102, P61983, P62260, P63039, Q66HD0, Q5XIM9, Q68FQ0
cytoskeletal protein (PC00085)	P34926, P0DJJ3, O35413, Q9WV63, P47819, B3GNI6, P63269, Q05764, P09495, Q9QYF3, P45592, B2GUZ5, P85108, P21575, Q68FR6, O35303, Q64122, P85515, Q4QRB4, Q5XI51, Q6P9T8, Q63279, P68035, P12839, Q64119, Q3T1K5, O70511, Q63610, Q5XI32, Q9Z1P2, P31000, P56536, P16409, Q68FR8, P52481, O08839, P70615, P19527, Q6AY56, Q5XIF6, Q9JJM9, P38650, Q3KRE8, P15205, Q4FZT0, D3ZHV2, Q6IG00, P70566, Q6QLM7, P30427, P16086, Q9WVC0, Q9JLT0, O08838, Q9WU82, P68370, P69897, Q07266, P13832, P48679, Q68FP1, Q4V7C7, Q9QWN8, Q9WU34, Q6KC51, Q2TA68, P60711, Q6AYH5, Q2PQA9, Q62812, Q9Z327, Q793F9, P23565, Q63028, Q6AYZ1, P09951, Q68FR8, P02600, Q63357, Q63537
dehydrogenase (PC00176	P11960, P04636, P29147, O70351, P04642, P19234, Q64428, O08651, Q9WVK7, Q641Y2, P41565, Q68FX0, Q99NA5, P21913, Q63342, P26284, P49432, P04797, Q5BK63, P12785, Q66HF1
enzyme modulator (PC00095)	O35413, B3GNI6, F1M386, Q9QYF3, Q63228, Q8R491, P62630, P61227, P21575, O35303, P08644, P10824, Q5PPJ9, P29066, P49911, P61751, P54311, P59215, P67874, P82471, P05197, Q9QUH6, O08839, P50399, Q9JJM9, P06238, Q9JIR4, P97846, P19627, Q9WVC0, Q8CGU4, Q9JLT0, O08838, Q6RUV5, Q6PST4, P85834, P01026, P43425, O35179, O35353, Q7TP98, Q9WVE9, Q9WU34, Q2TA68, Q62848, Q9ERH3, P97829, P04897, Q4FZT9, Q62812, Q63416, P52287, P61980, Q9Z1T4, Q63357
heterotrimeric G-protein (PC00095)	P10824, P54311, P59215, P82471, P19627, Q6PST4, P43425, O35353, P04897, P52287
hydrolase (PC00121)	P15999, P06687, Q06647, Q62950, P62630, P21575, O35303, P32551, Q62951, O08565, Q794F9, P11507, Q07009, Q63198, Q5RJQ4, P13233, P54311, Q64620, D3ZGS3, P11505, B5DFN2, O35567, P25286, B0BND0, P05197, Q9WTT6, P06685, O54715, B2RYG6, Q6AYD3, P38650, P11506, P62815, P97846, P62138, P13596, P85834, Q4QQT4, Q6Q0N1, O35353, Q64542, P06686, Q7TP98, Q2TA68, P28492, Q64568, Q62910, Q9ERH3, P97685, P63329, P12346, P10719, P63081, Q9QUL6, P52287, Q64578, P12785, P47942, O88767
intermediate filament (PC00085)	P47819, Q63279, P12839, P31000, P70615, P19527, Q6IG00, P48679, P23565
ion channel (PC00068)	P15999, P06687, Q63226, P81155, P54287, P11507, P11505, P06685, Q9R1Z0, P11506, P62815, Q71RJ2, P19491, Q63269, Q64542, Q00960, P06686, P29994, Q64568, P10719, Q9Z2L0, Q64578, P19490
membrane traffic protein (PC00150)	P0DJJ3, Q3B7K9, Q8R491, P63025, P61765, P21707, P97610, Q9JK11, P54921, Q6RJR6, P07825, P08081, P11442, P61265, P29101, P08082, P32851, P52303, Q63666, Q9WVE9, Q64548, P62944, P85969, P09951, Q5BJS7, P63045, Q9Z270, Q05140, P62744, P47709, P60881, Q62876, Q63537
membrane trafficking regulatory protein (PC00150)	P0DJJ3, P61765, P21707, P97610, P07825, P29101, P09951, Q9Z270, P47709, Q62876, Q63537
microtubule family cytoskeletal protein (PC00085)	P34926, Q9WV63, P85108, P21575, O35303, Q4QRB4, Q5XI51, Q6P9T8, P56536, Q68FR8, Q6AY56, Q5XIF6, P38650, Q3KRE8, P15205, Q6QLM7, P68370, P69897, Q2TA68, Q6AYH5, Q2PQA9, Q793F9, Q6AYZ1, Q68FR8
non-motor actin binding protein (PC00085)	Q05764, P45592, B2GUZ5, Q3T1K5, Q5XI32, Q9Z1P2, D3ZHV2, P70566, P30427, P16086, Q07266, Q68FP1, Q9QWN8, Q9Z327, Q63028, P09951, Q63537
oxidoreductase (PC00176)	P11960, P04636, P29147, O70351, P21396, P04642, Q68FR6, P19234, Q64428, O08651, Q9WVK7, Q641Y2, P35171, P41565, P11240, O35244, Q68FX0, Q99NA5, P21913, P52504, Q920J4, P12075, Q63342, P00406, P97846, P26284, P49432, Q63716, P35704, P04797, Q5BK63, P12785, Q66HF1
small GTPase (PC00095)	B3GNI6, P61227, P21575, O35303, P08644, P61751, Q9JJM9, Q9WVC0, Q6RUV5, Q9WU34, Q2TA68

Panther Protein Class (term ID)	Uniprot accessions of mapped proteins
structural protein (PC00211)	Q99JD4, P47819, P60203, Q63279, P12839, P31000, P70615, P19527, Q6IG00, P02688, P48679, Q6KC51, P07722, P23565
transcription factor (PC00218)	P97536, Q6MG48, P86252, Q68A21, Q6AYD3, O88767
transmembrane receptor regulatory/adaptor protein (PC00226)	Q62696, P97838, P0C6S7, P31016, P18484, P97546, P97837, P97836
transporter (PC00227)	P15999, P06687, Q06647, P97700, Q63226, Q9JI12, P81155, Q05962, Q505J6, P54287, Q9JHY2, P61765, P11507, O35458, P13638, P11505, Q63965, Q6QIX3, P25286, P06685, P48768, O54715, D3ZAF6, O35921, P07340, Q9R1Z0, P11506, P62815, Q71RJ2, P97846, P19491, P69682, Q63269, Q62634, Q64542, Q00960, P06686, P29994, Q64568, B5D5N9, Q7TNJ2, P31596, Q9JI66, P10719, P63081, P24942, Q09073, Q9Z2L0, Q64578, P19490, P16036, P31647
tubulin (PC00085)	P85108, Q4QRB4, Q6P9T8, Q68FR8, Q6AY56, Q5XIF6, Q3KRE8, P68370, P69897, Q6AYZ1, Q68FR8

Table S3 – BRI2 interactors retrieve from online databases. The BRI2 interactors, as well as, the uniprot accession number, the species and the experimental evidence supporting the interactions, as well as the cell line or tissue where they were detected is indicated.

Gene	Protein	Uniprot accession number	Species	Experimental evidence	Cell line/Tissue	References
Adam7	Desintegrin and metalloproteinase domain-containing protein 7	O35227	*Mus Musculus*	Affinity Capture-MS	Mouse sperm	49
AMIGO1	Adhesion molecule with Ig-like domain 1	Q86WK6	*Homo sapiens*	Affinity Capture-MS	HEK293T cells	50
APP	Amyloid beta A4 protein	P05067	*Homo sapiens*	Two-hybrid<br>Co-IP	HEK293 cells stably expressing APP751, transfected with myc-tagged BRI2	7,20
ATF6B	cAMP response element-binding protein-related protein	Q99941	*Homo sapiens*	Affinity Capture-MS	HEK293T cells	50
B4GALNT1	UDP-N-acetyl-alpha-D-galactosamine:(N-acetylneuraminyl)-galactosylglucosylceramide N-acetylgalactosaminyltransferase (GalNAc-T)	Q00973	*Homo sapiens*	Affinity Capture-MS	HEK293T cells	50
BACE1	β-secretase β-amyloid protein converting enzyme 1	P56817	*Homo sapiens*	Co-IP	SH-SY5Y cells	51
Bcl2	Apoptosis regulator Bcl-2<br>(protein phosphatase 1, regulatory subunit 50)	P10415	*Mus musculus*	Co-IP	TS1 alpha beta cells	52
BTN2A2	Butyrophilin, subfamily 2, member A2	Q8WVV5	*Homo sapiens*	Affinity Capture-MS	HEK293T cells	50
CACNA2D1	Dihydropyridine-sensitive L-type, calcium channel alpha-2/delta subunit	P54289	*Homo sapiens*	Affinity Capture-MS	HEK293T cells	50
CCDC155	Protein KASH5	Q8N6L0	*Homo sapiens*	Two-hybrid	----	53
CHST12	Carbohydrate (chondroitin 4) sulfotransferase 12	Q9NRB3	*Homo sapiens*	Affinity Capture-MS	HEK293T cells	50
CREB3	Cyclic AMP-responsive element-binding protein 3	O43889	*Homo sapiens*	Two-hybrid;<br>Affinity Capture-MS	HeLa cells expressing GFP-tagged proteins	54,55
Csf1	Macrophage colony-stimulating factor 1	P07141	*Mus musculus*	Pull-down	M1 myeloid cells	56
DCBLD2	Discoidin, CUB and LCCL domain-containing protein 2	Q96PD2	*Homo sapiens*	Affinity Capture-MS	HEK293T cells	50
FCGRT	IgG receptor FcRn large subunit p51	P55899	*Homo sapiens*	Affinity Capture-MS	HEK293T cells	50
GLG1	Cysteine-rich fibroblast growth factor receptor	Q92896	*Homo sapiens*	Affinity Capture-MS	HEK293T cells	50
HLA-A	HLA class I histocompatibility antigen, A-1 alpha chain	P30443	*Homo sapiens*	Affinity Capture-MS	HEK293T cells	50

Gene	Protein	Uniprot accession number	Species	Experimental evidence	Cell line/Tissue	References
HNF1A	Hepatocyte nuclear factor 1-alpha	P20823	*Homo sapiens*	Cross-linking study	Liver and pancreas	57
HS6ST1	Heparan sulfate 6-O-sulfotransferase 1	O60243	*Homo sapiens*	Affinity Capture-MS	HEK293T cells	50
KIAA1467	Uncharacterized protein KIAA1467	A2RU67	*Homo sapiens*	Affinity Capture-MS	HEK293T cells	50
KIF18A	Kinesin-like protein KIF18A	Q8NI77	*Homo sapiens*	Affinity Capture-MS	HeLa cells	55
Lmo2	Rhombotin-2	P25801	*Mus musculus*	Two-hybrid	----	58
LRFN3	Leucine-rich repeat and fibronectin type-III domain-containing protein 3	Q9BTN0	*Homo sapiens*	Affinity Capture-MS	HEK293T cells	50
MR1	Bajor histocompatibility complex class I-related gene protein	Q95460	*Homo sapiens*	Affinity Capture-MS	HEK293T cells	50
NAALADL2	Inactive N-acetylated-alpha-linked acidic dipeptidase-like protein 2	Q58DX5	*Homo sapiens*	Two-hybrid	----	53
Nek2	Serine/threonine-protein kinase Nek2	O35942	BRI2 *Hs* - Nek2 *Ms*	Affinity Capture-MS	HeLa cells	55
PGAP1	Post-GPI attachment to proteins factor 1	Q75T13	*Homo sapiens*	Affinity Capture-MS	HEK293T cells	50
PPP1CA/Ppp1ca	Serine/threonine-protein phosphatase PP1-alpha catalytic subunit	P62136	*Homo sapiens*	Co-IP	SH-SY5Y cells transfected with myc-bri2	5
		P62138	*Rattus norvergicus*	Co-IP	Rat primary cortical and hippocampal cultures	
PPP1CC/Ppp1cc	Serine/threonine-protein phosphatase PP1-gamma catalytic subunit	P36873	*Homo sapiens*	Co-IP	SH-SY5Y cells transfected with myc-bri2	5
		P63088	*Rattus norvergicus*	Co-IP	Rat primary cortical and hippocampal cultures	
RPL31	**60S ribosomal protein L31**	P62899	*Homo sapiens*	Two-hybrid	----	59
RYK	RYK receptor-like tyrosine kinase	P34925	*Homo sapiens*	Affinity Capture-MS	HEK293T cells	60
SEMA4F	Semaphorin-4F	O95754	*Homo sapiens*	Affinity Capture-MS	HEK293T cells	50
SEMA6A	Semaphorin-6A	Q9H2E6	*Homo sapiens*	Affinity Capture-MS	HEK293T cells	50
Shoc2	Leucine-rich repeat protein SHOC-2	O88520	BRI2 *Hs* - SHOC2 *Ms*	Affinity Capture-MS	HeLa cells expressing GFP-tagged proteins	55
SPPL2A	Signal peptide peptidase-like 2A	Q8TCT8	*Homo sapiens*	Co-IP	HEK293T cells	61

Gene	Protein	Uniprot accession number	Species	Experimental evidence	Cell line/Tissue	References
SPPL2B	Signal peptide peptidase-like 2B	Q8TCT7	*Homo sapiens*	Co-IP	HEK293T cells	[61]
SYNE4	Nesprin-4	Q8N205	*Homo sapiens*	Two-hybrid	----	[53]
TGFBR3	Transforming growth factor beta receptor type 3	Q03167	*Homo sapiens*	Affinity Capture-MS	HEK293T cells	[50]
TMEM17	Transmembrane protein 17	Q86X19	*Homo sapiens*	Proximity Label-MS	----	[62]
TMEM219	Insulin-like growth factor binding protein-3 receptor	Q86XT9	*Homo sapiens*	Affinity Capture-MS	HEK293T cells	[50]
TMEM59L	Brain-specific membrane-anchored protein (C19orf4)	Q9UK28	*Homo sapiens*	Affinity Capture-MS	HEK293T cells	[50]
UBC	Polyubiquitin-C	P0CG48	*Homo sapiens*	Affinity Capture-MS	HEK293T cells expressing HA-tagged ubiquitin; HEK293T cells expressing strep-HA tagged ubiquitin; HEK293T and HCT116 cells	[63–65]
UBR1	Ubiquitin protein ligase E3 component n-recognin 1	Q8IWV7	*Homo sapiens*	Affinity Capture-MS	HEK293T cells	[50]
UNK	RING finger protein unkempt homolog	Q9C0B0	*Homo sapiens*	Affinity Capture-RNA	SH-SY5Y and HeLa cells	[66]

Co-IP, co-immunoprecipitation; MS, mass spectrometry; Hs, *Homo sapiens*; Ms, *Mus musculus*.

Table S4 – Panther Protein class analysis of candidate BRI2 interactors identified by Nano-HPLC-MS/MS, and highly enriched or specific in the brain tissue. Panther protein class terms and the respective IDs, as well as the Uniprot accessions of the proteins that belong to each class are listed.

Panther Protein Class (term ID)	Uniprot accessions of mapped proteins
cation transporter (PC00227)	P06687, Q9JI12, P13638, P48768, O35921, P11506, Q62634, P06686, Q64568, P31596, P24942, P31647
cytoskeletal protein (PC00085)	P34926, P0DJJ3, P47819, Q05764, P85108, P21575, Q4QRB4, P12839, P56536, P19527, Q9JJM9, Q3KRE8, P15205, P70566, Q6QLM7, O08838, Q9QWN8, Q9WU34, P23565, P09951, Q63537
membrane traffic protein (PC00150)	P0DJJ3, Q8R491, P61765, P21707, P97610, P07825, P61265, P29101, P32851, Q64548, P85969, P09951, Q5BJS7, Q05140, P47709, P60881, Q63537
membrane trafficking regulatory protein (PC00150)	P0DJJ3, P61765, P21707, P97610, P07825, P29101, P09951, P47709, Q63537
microtubule family cytoskeletal protein (PC00085)	P34926, P85108, P21575, Q4QRB4, P56536, Q3KRE8, P15205, Q6QLM7, Q63537
myelin protein (PC00211)	P60203, P02688, P07722
nucleic acid binding(PC00022)	Q505J6, Q8CGU4
structural protein (PC00129)	P47819, P60203, P12839, P19527, P02688, P07722, P23565
transmembrane receptor regulatory/adaptor protein(PC00226)	P97838, P0C6S7, P31016, P97837, P97836
transporter(PC00227)	P06687, Q9JI12, Q505J6, P61765, O35458, P13638, Q6QIX3, P48768, O35921, P11506, Q71RJ2, P97846, P19491, Q62634, Q00960, P06686, Q64568, P31596, P24942, P19490, P31647
tubulin(PC00085)	P85108, Q4QRB4, Q3KRE8

Table S5 – Biological process enrichment analysis of the brain specific BRI2 interactome using Panther online resource. Enriched categories are identified as those with p value <0.05, and GO terms presented correspond to the two most specific for each category retrieved in the analysis. Nr. Number.

Biological Process	GO term	Nr. proteins	Fold enrichment (%)	p-value	Associated proteins
**Brain development (GO:0007420)**	brain development (GO:0007420)	31	7.39	1.31E-14	Slc17a6, Plp1, Ntm, Bcan, Syt1, Basp1, Cntn1, Nefm, Cnp, Gnao1, Slc32a1, Grin1, Dclk1, Slc8a2, Nefl, Atp2b2, Dcx, Mbp, Cend1, Ncam1, Kcna1, Slc17a7, Grin2b, Sptbn2, Mag, Atp2b3, Tnr, Slc1a2, Ina, Rph3a, Slc6a11
	forebrain development (GO:0030900)	16	7.07	1.02E-05	Slc17a6, Bcan, Nefm, Cnp, Gnao1, Slc32a1, Grin1, Dclk1, Slc8a2, Nefl, Dcx, Ncam1, Kcna1, Grin2b, Tnr, Slc1a2
	substantia nigra development (GO:0021762)	6	25.51	1.42E-03	Plp1, Basp1, Cnp, Mbp, Mag, Ina
	telencephalon development (GO:0021537)	12	7.99	3.69E-04	Slc17a6, Bcan, Nefm, Slc32a1, Grin1, Slc8a2, Nefl, Dcx, Kcna1, Grin2b, Tnr, Slc1a2
	hippocampus development (GO:0021766)	8	14.80	7.56E-04	Slc17a6, Bcan, Nefm, Slc32a1, Nefl, Dcx, Kcna1, Grin2b
**Neuron development (GO:0048666)**	positive regulation of dendrite extension (GO:1903861)	4	42.07	2.51E-02	Syt1, Syt2, Rims1, Cpne9
	regulation of dendrite extension (GO:1903859)	4	39.97	3.06E-02	Syt1, Syt2, Rims1, Cpne9
	neuron projection regeneration (GO:0031102)	5	24.98	1.75E-02	Gfap, Nefm, Nefl, Map1b, Ncam1
	neuron projection development (GO:0031175)	31	10.22	1.33E-18	Gfap, Crmp1, Plp1, Dpysl4, Tubb3, Stxbp1, Ncdn, Cntn1, Nefm, Dlg4, Cnp, Gnao1, Dclk1, Kif5c, Map6, Slc12a5, Nefl, Map1b, Dcx, Rab3a, Kif5a, Map2, Cntnap1, Agap2, Ncam1, Atl1, Gap43, Shank1, Snap91, Snap25, Camk2a
	dendrite morphogenesis (GO:0048813)	7	23.71	2.12E-04	Dlg4, Dclk1, Map6, Dcx, Map2, Shank1, Camk2a
	dendrite development (GO:0016358)	9	16.65	4.23E-05	Dlg4, Dclk1, Map6, Slc12a5, Map1b, Dcx, Map2, Shank1, Camk2a
	neuron projection morphogenesis (GO:0048812)	24	11.08	2.40E-14	Crmp1, Dpysl4, Tubb3, Stxbp1, Dlg4, Cnp, Dclk1, Kif5c, Map6, Nefl, Map1b, Dcx, Rab3a, Kif5a, Map2, Cntnap1, Agap2, Ncam1, Atl1, Gap43, Shank1, Snap91, Snap25, Camk2a
	cell morphogenesis involved in neuron differentiation (GO:0048667)	23	11.58	5.08E-14	Crmp1, Tubb3, Stxbp1, Dlg4, Cnp, Dclk1, Kif5c, Map6, Map1b, Atp2b2, Dcx, Rab3a, Kif5a, Map2, Agap2, Ncam1, Atl1, Gap43, Shank1, Slc1a3, Snap91, Snap25, Camk2a
	axonogenesis (GO:0007409)	17	10.26	9.76E-09	Crmp1, Tubb3, Stxbp1, Cnp, Dclk1, Kif5c, Map1b, Dcx, Rab3a, Kif5a, Map2, Agap2, Ncam1, Atl1, Gap43, Snap91, Snap25
	axon development (GO:0061564)	20	10.95	2.51E-11	Crmp1, Plp1, Tubb3, Stxbp1, Nefm, Cnp, Dclk1, Kif5c, Nefl, Map1b, Dcx, Rab3a, Kif5a, Map2, Agap2, Ncam1, Atl1, Gap43, Snap91, Snap25
**Regulation of synaptic plasticity (GO:0048167)**	positive regulation of excitatory postsynaptic potential (GO:2000463)	6	42.82	6.87E-05	Dlg4, Grin1, Stx1b, Rims1, Stx1a, Shank1
	modulation of excitatory postsynaptic potential (GO:0098815)	6	31.55	4.12E-04	Dlg4, Grin1, Stx1b, Rims1, Stx1a, Shank1
	positive regulation of synaptic transmission (GO:0050806)	17	24.98	5.86E-15	Gfap, Syt1, Syt12, Dlg4, Grin1, Stx1b, Slc8a2, Rims1, Gria2, Stx1a, Grin2b, Tnr, Shank1, Slc1a3, Camk2b, Snap25, Gria1
	neuron-neuron synaptic transmission (GO:0007270)	7	19.98	6.70E-04	Dnm1, Grin1, Tmod2, Slc17a7, Dlgap2, Napb, Gria1
	regulation of neuronal synaptic plasticity (GO:0048168)	11	30.53	1.28E-09	Bcan, Ncdn, Dlg4, Syp, Grin1, Slc8a2, Rab3a, Rims1, Grin2b, Camk2b, Camk2a
	regulation of long-term neuronal synaptic plasticity (GO:0048169)	6	34.26	2.55E-04	Dlg4, Syp, Grin1, Rims1, Grin2b, Camk2b
	long-term synaptic potentiation (GO:0060291)	8	31.97	2.03E-06	Gfap, Syt12, Slc8a2, Rims1, Grin2b, Tnr, Camk2b, Snap25
	long-term memory (GO:0007616)	6	33.31	3.01E-04	Grin1, Slc17a7, Grin2b, Shank1, Snap25, Gria1

Biological Process	GO term	Nr. proteins	Fold enrichment (%)	p-value	Associated proteins
**Learning or memory (GO:0007611)**	memory (GO:0007613)	8	12.02	3.60E-03	Atp1a3, Grin1, Slc8a2, Slc17a7, Grin2b, Shank1, Snap25, Gria1
	associative learning (GO:0008306)	7	14.57	5.49E-03	Atp1a3, Grin1, Grin2b, Atp1a2, Tnr, Shank1, Snap25
	learning (GO:0007612)	10	12.26	1.03E-04	Atp1a3, Grin1, Slc12a5, Slc8a2, Amph, Grin2b, Atp1a2, Tnr, Shank1, Snap25
**Synaptic vesicle cycle (GO:0099504)**	vesicle-mediated transport in synapse (GO:0099003)	15	26.76	2.71E-13	Pclo, Syt1, Syt12, Stx1b, Syt2, Cadps, Rab3a, Rims1, Amph, Stx1a, Sh3gl2, Cplx1, Sptbn2, Rph3a, Snap25
	neurotransmitter uptake (GO:0001504)	4	57.10	7.53E-03	Slc17a6, Atp1a2, Sv2a, Sv2b
	neurotransmitter transport (GO:0006836)	27	36.71	4.53E-30	Slc17a6, Pclo, Stxbp1, Syt1, Syt12, Slc32a1, Stx1b, Syt2, Cadps, Rab3a, Rims1, Stx1a, Slc17a7, Cplx1, Atp1a2, Sptbn2, Sv2a, Sv2b, Slc1a2, Slc1a3, Syn1, Nsf, Snap91, Rph3a, Snap25, Syn2, Slc6a11
	import into cell (GO:0098657)	5	21.72	3.43E-02	Slc17a6, Slc8a2, Atp1a2, Sv2a, Sv2b
	vesicle docking (GO:0048278)	6	23.51	2.29E-03	Stxbp1, Syt1, Stx1b, Stx1a, Sptbn2, Nsf
	membrane docking (GO:0022406)	6	17.90	1.10E-02	Stxbp1, Syt1, Stx1b, Stx1a, Sptbn2, Nsf
	regulation of synaptic vesicle priming (GO:0010807)	5	> 100	3.27E-06	Stxbp1, Stx1b, Rims1, Stx1a, Napb
	regulation of protein complex assembly (GO:0043254)	11	6.16	1.73E-02	Add2, Stxbp1, Stx1b, Gda, Map1b, Rims1, Tmod2, Ncam1, Stx1a, Sptbn2, Napb
	vesicle fusion (GO:0006906)	7	15.54	3.58E-03	Syt12, Stx1b, Syt2, Stx1a, Nsf, Rph3a, Snap25
	organelle membrane fusion (GO:0090174)	7	15.21	4.15E-03	Syt12, Stx1b, Syt2, Stx1a, Nsf, Rph3a, Snap25
	single-organism organelle organization (GO:1902589)	28	3.86	4.44E-06	Map1a, Gfap, Add2, Crmp1, Ehd3, Stxbp1, Syt12, Nefm, Dlg4, Cnp, Stx1b, Map6, Nefl, Syt2, Map1b, Rab3a, Tmod2, Klc1, Map2, Stx1a, Slc17a7, Shank1, Ina, Slc1a3, Nsf, Rph3a, Snap25, Camk2a
	vesicle organization (GO:0016050)	13	10.82	2.71E-06	Dnm1, Stxbp1, Syt12, Dlg4, Stx1b, Syt2, Cadps, Rab3a, Stx1a, Slc17a7, Nsf, Rph3a, Snap25
	regulation of synaptic vesicle exocytosis (GO:2000300)	6	54.50	1.66E-05	Stxbp1, Stx1b, Rab3a, Rims1, Stx1a, Napb
	synaptic vesicle exocytosis (GO:0016079)	13	35.59	1.03E-12	Pclo, Syt1, Syt12, Stx1b, Syt2, Cadps, Rab3a, Rims1, Stx1a, Cplx1, Sptbn2, Rph3a, Snap25
	calcium ion-regulated exocytosis of neurotransmitter (GO:0048791)	7	35.87	1.27E-05	Syt1, Syt12, Stx1b, Syt2, Rims1, Rph3a, Snap25
	positive regulation of calcium ion-dependent exocytosis (GO:0045956)	6	54.50	1.66E-05	Scamp5, Stxbp1, Syt1, Cadps, Rims1, Stx1a
	positive regulation of regulated secretory pathway (GO:1903307)	7	28.55	6.02E-05	Scamp5, Stxbp1, Syt1, Cadps, Rab3a, Rims1, Stx1a
	protein localization to synapse (GO:0035418)	7	77.72	6.17E-08	Homer1, Pclo, Dlg4, Klc1, Shank1, Bsn, Dlgap1
	protein localization (GO:0008104)	28	3.58	2.30E-05	Scamp5, Ehd3, Homer1, Pclo, Dlg2, Stxbp1, Dlg4, Kif5c, Stx1b, Cadps, Rab3a, Rims1, Kif5a, Klc1, Cntnap1, Gria2, Stx1a, Kcna1, Cplx1, Grin2b, Shank1, Napb, Nsf, Snap91, Rph3a, Bsn, Rab3c, Dlgap1
	synaptic vesicle maturation (GO:0016188)	4	88.82	1.31E-03	Stxbp1, Dlg4, Rab3a, Slc17a7
	developmental maturation (GO:0021700)	10	9.25	1.38E-03	Plp1, Dlg2, Stxbp1, Dlg4, Grin1, Map1b, Rab3a, Cend1, Slc17a7, Shank1
	synaptic vesicle endocytosis (GO:0048488)	5	52.59	4.59E-04	Syt1, Syt12, Syt2, Amph, Sh3gl2

Biological Process	GO term	Nr. proteins	Fold enrichment (%)	p-value	Associated proteins
	clathrin-dependent endocytosis (GO:0072583)	6	38.68	1.25E-04	Dnm1, Syt1, Syt12, Syt2, Amph, Sh3gl2
	synaptic vesicle recycling (GO:0036465)	6	47.96	3.53E-05	Rab3a, Syt1, Syt12, Syt2, Amph, Sh3gl2
**Ion transport (GO:0006811)**	inorganic cation transmembrane transport (GO:0098662)	13	5.42	7.99E-03	Atp1a3, Atp1b2, Grin1, Slc30a3, Slc12a5, Slc8a2, Atp2b2, Cacng2, Kcna1, Slc17a7, Atp1a2, Atp2b3, Snap25
	cation transport (GO:0006812)	19	4.81	1.36E-04	Atp1a3, Slc17a6, Slc32a1, Atp1b2, Grin1, Slc30a3, Slc12a5, Slc8a2, Atp2b2, Cacng2, Kcna1, Slc17a7, Grin2b, Atp1a2, Atp2b3, Nsf, Camk2b, Snap25, Camk2a
	metal ion transport (GO:0030001)	18	6.48	3.42E-06	Atp1a3, Slc17a6, Atp1b2, Grin1, Slc30a3, Slc12a5, Slc8a2, Atp2b2, Cacng2, Kcna1, Slc17a7, Grin2b, Atp1a2, Atp2b3, Nsf, Camk2b, Snap25, Camk2a
	regulation of ion transport (GO:0043269)	15	4.65	7.61E-03	Ehd3, Homer1, Syt1, Cntn1, Dlg4, Gnao1, Atp1b2, Grin1, Atp2b2, Cacng2, Kcna1, Grin2b, Atp1a2, Shank1, Camk2a
**L-glutamate transport (GO:0015813)**	L-glutamate transmembrane transport (GO:0089711)	4	72.67	2.90E-03	Slc17a6, Slc1a6, Slc1a2, Slc1a3
	L-glutamate transport (GO:0015813)	5	55.51	3.52E-04	Slc17a6, Slc1a6, Slc1a2, Slc1a3, Slc17a7
**Glutamate receptor signalling pathway (GO:0007215)**	ionotropic glutamate receptor signaling pathway (GO:0035235)	6	46.12	4.44E-05	Atp1a3, Grin1, Gria2, Grin2b, Gria1, Camk2a
	glutamate receptor signaling pathway (GO:0007215)	7	31.79	2.89E-05	Atp1a3, Grin1, Gria2, Grin2b, Gria1, Camk2a, Homer1
**Neuromuscular process (GO:0050905)**	neuromuscular process controlling balance (GO:0050885)	8	24.60	1.55E-05	Dlg4, Nefl, Atp2b2, Cntnap1, Tnr, Shank1, Slc1a3, Camk2b
	neuromuscular process (GO:0050905)	11	18.32	2.88E-07	Dlg4, Grin1, Nefl, Atp2b2, Cntnap1, Kcna1, Grin2b, Tnr, Shank1, Slc1a3, Camk2b
**Behavior (GO:0007610)**	locomotory behavior (GO:0007626)	12	10.61	1.68E-05	Atp1a3, Dnm1, Dlg4, Cnp, Gnao1, Grin1, Atp2b2, Cend1, Lsamp, Atp1a2, Tnr, Snap25
	adult behavior (GO:0030534)	10	11.69	1.61E-04	Atp1a3, Dnm1, Homer1, Cnp, Grin1, Cend1, Atp1a2, Sptbn2, Slc1a2, Shank1
**Response to chemical (GO:0042221)**	response to fungicide (GO:0060992)	4	39.97	3.06E-02	Grin1, Gria2, Grin2b, Gria1
	response to inorganic substance (GO:0010035)	15	4.65	7.46E-03	Homer1, Syt1, Gnao1, Grin1, Slc30a3, Nefl, Map1b, Mbp, Gria2, Ncam1, Kcna1, Grin2b, Slc1a3, Camk2b, Gria1
**Others**	microtubule-based process (GO:0007017)	14	4.98	8.14E-03	Map1a, Crmp1, Tubb2a, Tubb3, Nefm, Cnp, Kif5c, Map6, Nefl, Tubb2b, Map1b, Kif5a, Klc1, Map2
	cytoskeleton organization (GO:0007010)	20	4.42	2.15E-04	Map1a, Gfap, Add2, Crmp1, Tubb2a, Pclo, Tubb3, Nefm, Cnp, Map6, Nefl, Tubb2b, Map1b, Tmod2, Map2, Cntnap1, Sptbn2, Shank1, Ina, Bsn
	synapse organization (GO:0050808)	10	12.34	9.71E-05	Pclo, Dlg4, Map1b, Atp2b2, Rab3a, Cacng2, Sptbn2, Tnr, Shank1, Bsn
	sensory perception of mechanical stimulus (GO:0050954)	9	10.77	1.67E-03	Map1a, Dnm1, Atp2b2, Rab3a, Mbp, Kcna1, Grin2b, Slc1a3, Crym
	negative regulation of cellular component organization (GO:0051129)	16	5.09	9.51E-04	Scamp5, Map1a, Gfap, Add2, Crmp1, Ntm, Stxbp1, Dlg4, Stx1b, Map1b, Mbp, Tmod2, Sptbn2, Mag, Tnr, Shank1

Table S6 – Cellular component enrichment analysis of the brain specific BRI2 interactome using Panther online resource. Enriched categories are identified as those with p value <0.05, and GO terms presented correspond to the two most specific for each category retrieved in the analysis. Nr. Number.

GO term	Nr. proteins	Fold enrichment (%)	p-value	Associated proteins
synaptobrevin 2-SNAP-25-syntaxin-1a-complexin I complex (GO:0070032)	3	> 100	1.61E-03	Stx1a, Cplx1, Snap25
SNARE complex (GO:0031201)	8	31.35	3.57E-07	Scamp5, Syt1, Stx1b, Stx1a, Cplx1, Napb, Snap25, Syn2
synaptobrevin 2-SNAP-25-syntaxin-1a complex (GO:0070044)	3	> 100	3.14E-03	Stx1a, Napb, Snap25
neurofilament (GO:0005883)	4	79.94	3.00E-04	Nefm, Nefl, Dlgap2, Ina
polymeric cytoskeletal fiber (GO:0099513)	19	6.67	9.85E-08	Map1a, Gfap, Crmp1, Tubb2a, Dnm1, Tubb3, Nefm, Cnp, Kif5c, Map6, Slc8a2, Nefl, Tubb2b, Map1b, Dcx, Kif5a, Klc1, Map2, Dlgap2, Ina
NMDA selective glutamate receptor complex (GO:0017146)	4	66.61	6.17E-04	Dlgap3, Grin1, Grin2b, Shank1
cation channel complex (GO:0034703)	11	12.63	2.06E-06	Dlgap3, Dlg2, Dlg4, Grin1, Cacng2, Cntnap1, Stx1a, Kcna1, Grin2b, Shank1, Snap25
ionotropic glutamate receptor complex (GO:0008328)	9	35.97	7.92E-09	Dlgap3, Dlg2, Dlg4, Grin1, Cacng2, Gria2, Grin2b, Shank1, Gria1
excitatory synapse (GO:0060076)	12	66.61	1.49E-15	Slc17a6, Ntm, Homer1, Syt1, Dlg4, Syp, Grin1, Stx1b, Slc17a7, Shank1, Bsn, Gria1
synapse (GO:0045202)	69	15.72	2.70E-63	Scamp5, Atp1a3, Map1a, Srcin1, Sgip1, Add2, Slc17a6, Dnm1, Ntm, Bcan, Dlgap3, Homer1, Pclo, Dlg2, Stxbp1, Syt1, Anks1b, Syt12, Nefm, Dlg4, Prrt1, Syp, Slc32a1, Grin1, Gabbr2, Slc30a3, Dclk1, Stx1b, Slc8a2, Syt2, Sept5, Map1b, Atp2b2, Prkcg, Rab3a, Rims1, Map2, Amph, Gria2, Stx1a, Kcna1, Sh3gl2, Slc17a7, Cplx1, Grin2b, Atp1a2, Sptbn2, Sept3, Sv2a, Sv2b, Gap43, Slc1a2, Dlgap2, Shank1, Slc1a3, Syn1, Nsf, Snap91, Camk2b, Rph3a, Snap25, Bsn, Cnksr2, Gria1, Syn2, Camk2a, Rab3c, Dlgap1
juxtaparanode region of axon (GO:0044224)	3	66.61	1.80E-02	Dlg2, Dlg4, Kcna1
main axon (GO:0044304)	9	24.30	2.44E-07	Dnm1, Dlg2, Dlg4, Map1b, Mbp, Cntnap1, Kcna1, Mag, Slc1a2
ciliary rootlet (GO:0035253)	3	59.95	2.47E-02	Kif5a, Kif5c, Klc1
sodium:potassium-exchanging ATPase complex (GO:0005890)	3	59.95	2.47E-02	Atp1a3, Atp1b2, Atp1a2
synaptic vesicle membrane (GO:0030672)	16	50.75	1.54E-19	Scamp5, Slc17a6, Syt1, Syt12, Syp, Slc30a3, Syt2, Amph, Gria2, Stx1a, Slc17a7, Sv2a, Sv2b, Syn1, Rph3a, Syn2
synaptic vesicle (GO:0008021)	29	37.15	1.47E-33	Scamp5, Slc17a6, Dnm1, Syt1, Syt12, Dlg4, Syp, Slc32a1, Grin1, Slc30a3, Stx1b, Syt2, Sept5, Rab3a, Amph, Gria2, Stx1a, Slc17a7, Grin2b, Sptbn2, Sv2a, Sv2b, Syn1, Snap91, Rph3a, Snap25, Gria1, Syn2, Rab3c
presynaptic active zone (GO:0048786)	8	48.45	1.18E-08	Pclo, Syp, Slc32a1, Rims1, Slc17a7, Sv2a, Syn1, Bsn
presynaptic membrane (GO:0042734)	13	33.74	3.07E-13	Syt1, Syp, Stx1b, Rims1, Gria2, Stx1a, Kcna1, Grin2b, Sept3, Slc1a2, Snap91, Snap25, Camk2a
synaptic membrane (GO:0097060)	27	19.76	7.71E-24	Srcin1, Dlgap3, Homer1, Dlg2, Syt1, Anks1b, Dlg4, Syp, Grin1, Gabbr2, Stx1b, Prkcg, Rims1, Gria2, Stx1a, Kcna1, Grin2b, Sept3, Slc1a2, Dlgap2, Shank1, Snap91, Snap25, Cnksr2, Gria1, Camk2a, Dlgap1
terminal bouton (GO:0043195)	19	32.45	5.42E-20	Sgip1, Pclo, Stxbp1, Syt1, Syp, Grin1, Syt2, Sept5, Rab3a, Amph, Gria2, Cplx1, Grin2b, Sv2a, Sv2b, Syn1, Snap91, Snap25, Syn2
axon terminus (GO:0043679)	21	21.86	7.62E-19	Sgip1, Pclo, Stxbp1, Syt1, Syp, Slc32a1, Grin1, Syt2, Sept5, Rab3a, Amph, Gria2, Kcna1, Cplx1, Grin2b, Sv2a, Sv2b, Syn1, Snap91, Snap25, Syn2
AMPA glutamate receptor complex (GO:0032281)	4	26.65	2.25E-02	Dlg4, Cacng2, Gria2, Gria1

GO term	Nr. proteins	Fold enrichment (%)	p-value	Associated proteins
postsynaptic density (GO:0014069)	31	26.14	1.76E-31	Map1a, Srcin1, Add2, Dlgap3, Homer1, Pclo, Dlg2, Anks1b, Nefm, Dlg4, Grin1, Dclk1, Map1b, Prkcg, Rims1, Map2, Gria2, Grin2b, Gap43, Dlgap2, Shank1, Syn1, Nsf, Snap91, Camk2b, Bsn, Cnksr2, Gria1, Syn2, Camk2a, Dlgap1
postsynaptic specialization (GO:0099572)	31	26.14	1.76E-31	Map1a, Srcin1, Add2, Dlgap3, Homer1, Pclo, Dlg2, Anks1b, Nefm, Dlg4, Grin1, Dclk1, Map1b, Prkcg, Rims1, Map2, Gria2, Grin2b, Gap43, Dlgap2, Shank1, Syn1, Nsf, Snap91, Camk2b, Bsn, Cnksr2, Gria1, Syn2, Camk2a, Dlgap1
asymmetric synapse (GO:0032279)	31	25.60	3.30E-31	Map1a, Srcin1, Add2, Dlgap3, Homer1, Pclo, Dlg2, Anks1b, Nefm, Dlg4, Grin1, Dclk1, Map1b, Prkcg, Rims1, Map2, Gria2, Grin2b, Gap43, Dlgap2, Shank1, Syn1, Nsf, Snap91, Camk2b, Bsn, Cnksr2, Gria1, Syn2, Camk2a, Dlgap1
dendritic shaft (GO:0043198)	7	23.31	3.58E-05	Homer1, Slc12a5, Map2, Gria2, Slc1a2, Nsf, Gria1
dendrite (GO:0030425)	37	11.83	4.31E-26	Atp1a3, Srcin1, Crmp1, Bcan, Dlgap3, Homer1, Pclo, Tubb3, Dlg2, Anks1b, Ncdn, Dlg4, Gnao1, Slc32a1, Grin1, Slc12a5, Slc8a2, Map1b, Atp2b2, Prkcg, Dcx, Map2, Gnaz, Gria2, Kcna1, Cplx1, Grin2b, Atp1a2, Slc1a2, Dlgap2, Shank1, Slc1a3, Nsf, Camk2b, Bsn, Gria1, Camk2a
myelin sheath (GO:0043209)	23	22.10	5.60E-21	Atp1a3, Gfap, Ehd3, Plp1, Dnm1, Stxbp1, Cntn1, Nefm, Cnp, Gnao1, Nefl, Mbp, Cntnap1, Ncam1, Atp1a2, Mag, Ina, Mog, Napb, Syn1, Nsf, Snap25, Syn2
dendritic spine (GO:0043197)	14	18.17	8.97E-11	Atp1a3, Dlgap3, Anks1b, Dlg4, Grin1, Slc8a2, Map1b, Gria2, Grin2b, Atp1a2, Slc1a2, Shank1, Slc1a3, Gria1
neuron spine (GO:0044309)	14	17.93	1.07E-10	Atp1a3, Dlgap3, Anks1b, Dlg4, Grin1, Slc8a2, Map1b, Gria2, Grin2b, Atp1a2, Slc1a2, Shank1, Slc1a3, Gria1
postsynaptic membrane (GO:0045211)	16	16.15	8.02E-12	Srcin1, Dlgap3, Homer1, Dlg2, Anks1b, Dlg4, Grin1, Gabbr2, Gria2, Grin2b, Dlgap2, Shank1, Snap91, Cnksr2, Gria1, Dlgap1
voltage-gated potassium channel complex (GO:0008076)	6	15.78	3.41E-03	Dlg2, Dlg4, Cntnap1, Stx1a, Kcna1, Snap25
potassium channel complex (GO:0034705)	6	14.45	5.62E-03	Dlg2, Dlg4, Cntnap1, Stx1a, Kcna1, Snap25
growth cone (GO:0030426)	11	12.42	2.46E-06	Crmp1, Pclo, Basp1, Nefl, Map1b, Tmod2, Klc1, Gria2, Ncam1, Gap43, Snap25
site of polarized growth (GO:0030427)	11	12.08	3.27E-06	Crmp1, Pclo, Basp1, Nefl, Map1b, Tmod2, Klc1, Gria2, Ncam1, Gap43, Snap25
perikaryon (GO:0043204)	7	9.58	1.27E-02	Nefm, Slc12a5, Slc8a2, Map1b, Gria2, Kcna1, Camk2b
neuronal cell body (GO:0043025)	30	9.46	9.35E-18	Srcin1, Crmp1, Dlgap3, Homer1, Pclo, Tubb3, Dlg2, Ncdn, Nefm, Grin1, Slc12a5, Slc8a2, Map1b, Atp2b2, Mbp, Kif5a, Klc1, Map2, Gria2, Ncam1, Kcna1, Cplx1, Sptbn2, Slc1a3, Camk2b, Snap25, Bsn, Cnksr2, Gria1, Camk2a
microtubule (GO:0005874)	13	7.97	1.53E-05	Map1a, Tubb2a, Dnm1, Tubb3, Cnp, Kif5c, Map6, Slc8a2, Tubb2b, Map1b, Dcx, Kif5a, Klc1, Map2
cell junction (GO:0030054)	48	7.54	1.36E-26	Scamp5, Srcin1, Slc17a6, Ehd3, Dlgap3, Homer1, Pclo, Dlg2, Syt1, Basp1, Anks1b, Syt12, Dlg4, Prrt1, Syp, Grin1, Gabbr2, Slc30a3, Syt2, Map1b, Cadps, Atp2b2, Prkcg, Rims1, Amph, Gria2, Cadm4, Ncam1, Stx1a, Kcna1, Slc17a7, Grin2b, Sptbn2, Sept3, Sv2a, Sv2b, Gap43, Dlgap2, Shank1, Syn1, Rph3a, Snap25, Bsn, Gria1, Syn2, Camk2a, Dlgap1
Golgi apparatus (GO:0005794)	18	3.02	3.46E-02	Scamp5, Atp1a3, Itm2b, Dnm1, Pclo, Syt1, Map6, Slc1a6, Amph, Atl1, Sptbn2, Atp2b3, Syn1, Nsf, Rph3a, Snap25, Bsn, Cnksr2

Table S7 – Brain enriched/specific Nano-HPLC-MS/MS identified candidate BRI2 interacting proteins annotated with GO terms related to neuronal differentiation processes. Uniprot accession numbers and gene names are listed.

Uniprot Acession	Gene name	Uniprot Acession	Gene name
P21707	Syt1	Q63622	Dlg2
P29101	Syt2	P61765	Stxbp1
Q9JIR4	Rims1	O35095	Ncdn
Q5BJS7	Cpne9	P31016	Dlg4
Q9QXY2	Srcin1	P13233	Cnp
P47819	Gfap	P59215	Gnao1
Q62950	Crmp1	O08875	Dclk1
Q62718	Ntm	P56536	Kif5c
Q63198	Cntn1	Q63560	Map6
P12839	Nefm	Q63633	Slc12a5
P35439	Grin1	Q3KRE8	Tubb2b
P61265	Stx1b	P11506	Atp2b2
P19527	Nefl	P63012	Rab3a
P15205	Map1b	Q6QLM7	Kif5a
P02688	Mbp	P97846	Cntnap1
P15146	Map2	Q8CGU4	Agap2
P07722	Mag	P13596	Ncam1
Q05546	Tnr	Q6PST4	Atl1
Q9WV48	Shank1	P10499	Kcna1
P08413	Camk2b	Q64548	Rtn1
P60881	Snap25	P07936	Gap43
Q5FVI4	Cend1	P24942	Slc1a3
P60203	Plp1	Q05140	Snap91
Q62951	Dpysl4	P11275	Camk2a
Q4QRB4	Tubb3		
